# Defined chromosome structure in the genome-reduced bacterium *Mycoplasma pneumoniae*

**DOI:** 10.1038/ncomms14665

**Published:** 2017-03-08

**Authors:** Marie Trussart, Eva Yus, Sira Martinez, Davide Baù, Yuhei O. Tahara, Thomas Pengo, Michael Widjaja, Simon Kretschmer, Jim Swoger, Steven Djordjevic, Lynne Turnbull, Cynthia Whitchurch, Makoto Miyata, Marc A. Marti-Renom, Maria Lluch-Senar, Luís Serrano

**Affiliations:** 1 EMBL/CRG Systems Biology Research Unit, Centre for Genomic Regulation (CRG), The Barcelona Institute of Science and Technology, Dr Aiguader 88, Barcelona 08003, Spain; 2 Universitat Pompeu Fabra (UPF), 08003 Barcelona, Spain; 3 Gene Regulation, Stem Cells and Cancer Program. Centre for Genomic Regulation (CRG), The Barcelona Institute of Science and Technology, Dr Aiguader 88, Barcelona 08003, Spain; 4 CNAG-CRG, Centre for Genomic Regulation (CRG), The Barcelona Institute of Science and Technology, Baldiri Reixac 4, Barcelona 08028, Spain; 5 Department of Biology, Graduate School of Science, Osaka City University, 558-8585 Osaka, Japan; 6 OCU Advanced Research Institute for Natural Science and Technology (OCARNA), Osaka City University, 558-8585 Osaka, Japan; 7 Advanced Light Microscopy Unit, Centre for Genomic Regulation (CRG), 08003 Barcelona, Spain; 8 The ithree Institute, The University of Technology Sydney, Sydney, New South Wales 2007, Australia; 9 Department of Cellular and Molecular Biophysics, Max Planck Institute of Biochemistry, 82152, Martinsried, Germany; 10 Institució Catalana de Recerca i Estudis Avançats (ICREA), 08010 Barcelona, Spain

## Abstract

DNA-binding proteins are central regulators of chromosome organization; however, in genome-reduced bacteria their diversity is largely diminished. Whether the chromosomes of such bacteria adopt defined three-dimensional structures remains unexplored. Here we combine Hi-C and super-resolution microscopy to determine the structure of the *Mycoplasma pneumoniae* chromosome at a 10 kb resolution. We find a defined structure, with a global symmetry between two arms that connect opposite poles, one bearing the chromosomal Ori and the other the midpoint. Analysis of local structures at a 3 kb resolution indicates that the chromosome is organized into domains ranging from 15 to 33 kb. We provide evidence that genes within the same domain tend to be co-regulated, suggesting that chromosome organization influences transcriptional regulation, and that supercoiling regulates local organization. This study extends the current understanding of bacterial genome organization and demonstrates that a defined chromosomal structure is a universal feature of living systems.

## Abstract (web summary)

The three-dimensional architecture of genome-reduced bacteria is poorly understood. Here the authors combine Hi-C with super-resolution microscopy in *Mycoplasma pneumoniae* and provide evidence of how supercoiling and local organization influences gene regulation.

Several studies have revealed novel insights into chromatin dynamics and its effect on gene expression regulation and replication^1^,^2^. Such interplay suggests that chromatin organization might have a role in regulating gene expression at both the global and gene-specific levels^3^. In all kingdoms of life, genome organization occurs in a functional and dynamic manner, packing the genome into the nucleus in the case of eukaryotes, while packing it into the cell in the case of bacteria. Bacteria have evolved mechanisms such as DNA supercoiling^4^ and nucleoid-associated proteins (NAPs) mediated folding^5^ to condense their chromosomes. Negative supercoiling forms plectonemic loops of 10–100 kilobases (kb)^6^,^7^, which are maintained by both gyrases and topoisomerases^6^. Simultaneously, DNA-based processes such as transcription, replication and repair are all efficiently accommodated.

In the past, diffraction-limited resolution has impaired the detailed characterization of chromosome structures. However, more recent developments in super-resolution localization microscopy^8^,^9^,^10^,^11^,^12^ and chromosome conformation capture (3C)-based techniques^13^ have enabled the determination of the global chromosome organization of some bacteria^14^. High-throughput derivations of genome-wide 3C-based assays such as Hi-C technologies^15^ have been used to generate high-resolution contact maps of genomes, which when combined with modelling, can provide three-dimensional (3D) representations of genome structures^16^,^17^,^18^,^19^. Studies of bacterial chromosome organization and regulation using these afore mentioned combinatorial techniques have been carried out in *Caulobacter crescentus,* resulting in a Hi-C map with a 13 kb resolution^17^, in *Escherichia coli* with a 20 kb resolution^20^, and in *Bacillus subtilis* with 30, 10 and 4 kb resolutions^21^,^22^,^23^. These studies show that for *C. crescentus* the genome structure is globally related to the chromosome segregation process, while for *E. coli* it is more related to DNA replication and transcription. More recently, a higher 10 kb resolution Hi-C map of *C. crescentus*^18^ revealed that its genome is divided into 23 chromosome interacting domains (CIDs) or highly self-interacting regions, similar to the topologically associating domains (TADs) found in eukaryotes^24^,^25^. The size of these CIDs range from 30 to 400 kb in *C. crescentus*^18^, and from 50 to 300 kb (ref. 22) or 60 to 340 kb (ref. 23) in *B. subtilis*, depending on the study.

Nonetheless, in *C. crescentus,* the strongest determinant of these domain boundaries was the presence of highly expressed genes, whereas surprisingly the absence of the NAP heat unstable (HU) histone-like proteins and structural maintenance of chromosomes (SMC) proteins did not significantly affect the domain boundaries^18^. No such domains were described in the lower resolution Hi-C map of *E. coli*. Nevertheless, it was found that histone-like proteins such as factor for inversion simulation (Fis), integration host factor (IHF) and histone-like nucleoid structuring (H-NS) do not contribute to the global organization of the *E. coli* genome^20^. Additionally, on a larger scale, it has been shown that the *E. coli* genome consists of four macrodomain-like regions of about 1 megabase (Mb) each, and two less constrained regions, all of which influence the segregation and mobility of the chromosome^26^.

The above mentioned bacteria all have large, complex genomes over 4 Mb in size, coding for hundreds of transcription factors (TFs)^27^, multiple DNA structural proteins and several sigma factors, each of which play key roles in the response to physiological and environmental signals^28^. How such structural organization is achieved and the impact that it has on transcriptional regulation is unknown. Furthermore, whether smaller bacteria with reduced genomes and reduced total copy numbers of structural proteins maintain a defined chromosome structure is also undetermined. To address this question, we studied the chromosome organization of the genome-reduced bacterium, *Mycoplasma pneumoniae*, which has minimal genetic components and lacks several structural DNA-binding proteins^29^. It has been shown that changes in DNA supercoiling can control transcription in bacteria^30^, and in fact, this could be more important in small-genome bacteria such as Mycoplasmas^31^ where, despite the absence of many structural DNA-binding proteins^29^, both gyrases and topoisomerases are present to control gene expression through changing the local DNA structure^31^. *M. pneumoniae* is one of the smallest self-replicating organisms^32^, has no cell wall, and causes atypical pneumonia in humans^33^. It possesses an attachment organelle (AO) located at one of the cell poles that is involved in adherence, motility and cell division^34^. *M. pneumoniae* has only a few known NAPs, and the total copy number for the remaining proteins is smaller compared with other bacteria (Table 1 and Supplementary Data 1): MPN529, IHF-HU that possibly affects DNA topology^35^; MPN426, an SMC family protein; MPN229 and MPN554, proteins that bind single-stranded DNA (ssDNA)^36^,^37^; and MPN002 a possible homologue of CbpA. It also has very few TFs and only two sigma factors are found within its genome (Table 1)^38^. In addition, *M. pneumoniae* has been systematically characterized in a quantitative manner by transcriptomics, proteomics and metabolomics studies^39^,^40^,^41^,^42^,^43^,^44^.

Here, we used Hi-C to determine the 3D structure of the stationary *M. pneumoniae* chromosome at a 10 kb resolution, with local structures further resolved at 3 kb. This model is validated by electron microscopy, 3D light microscopy reconstruction of the nucleoid, and super-resolution microscopy of exponential and stationary cells. We observed a general symmetry along the axis of the origin (Ori) and the midpoint of the chromosome. The Ori and the midpoint are located at the two opposite poles of the chromosome structure. Moreover we detected that the chromosome is organized into 44 CIDs, ranging from 15 to 33 kb, which are smaller than the CIDs previously described for *C. crescentus*^18^ and *B. subtilis*^22^,^23^. Inhibiting supercoiling induced a decrease in the domain border strengths, suggesting that supercoiling might play a role in the regulation of these domains. Interestingly, we provide the first evidence that genes inside CIDs tend to be co-regulated, suggesting that chromosome organization could influence transcriptional regulation. Our results, together with previous 3D structures of other bacterial chromosomes and data on eukaryotes, indicate that chromosome organization in cells is a widespread phenomenon of life.

## Results

### From *M. pneumoniae* Hi-C maps to 3D chromatin structure

Here, we studied the *M. pneumoniae* genome organization during stationary phase, performing Hi-C using four-cutter, HpaII, and six-cutter, HindIII, enzymes with average cutting frequencies of 450 and 1,810 base pairs (bp), respectively. Hi-C experiments generate genome-wide libraries of ligation products, in which genomic loci that are positioned close in space contact frequently, and loci located far away rarely interact. Spatial distances can then be inferred from genomic loci frequencies to reflect the 3D structure of the genome. Although the Hi-C interaction maps obtained at exponential and stationary phase display similar features (Supplementary Fig. 6), the analysis of the exponential phase data alluded to possible chromosome heterogeneity. Therefore, as it is not possible to synchronize *M. pneumoniae,* we concentrated on the stationary phase samples for 3D modelling. To analyse the Hi-C data sets, the paired-end library reads were first uniquely mapped to the MPN129 reference genome (NC_000912, NCBI) covering 816,394 bp. This was done using Bowtie2, and further filtered and normalized as previously described following the ICE iterative mapping strategy from the hiclib Python^45^ (Methods). Briefly, read pairs were classified as valid Hi-C products, non-ligation products, or self-ligation products, and only the valid Hi-C products were considered. We then constructed a genome wide matrix, M, of different resolutions (3, 5, 10, 15 and 20 kb) by dividing the genome into 3, 5, 10, 15 and 20 kb bins respectively, and pooling interactions into their corresponding bins. The final interaction frequencies were represented as two-dimensional matrices, where *M(i,j)* indicates the relative frequency of interaction between fragments in bins *i* and *j*.

The decision to use the resolution of 10 kb was determined based on the correlation between 7 replicates at 3, 5, 10, 15 and 20 kb resolutions (Supplementary Figs 1 and 2), as well as on the matrix modelling potential (MMP) score of the resulting matrices (Supplementary Fig. 3 and Supplementary Table 1). The MMP score assesses the potential of an interaction matrix to determine accurate 3D models. At a 10 kb resolution, both the HindIII and HpaII data sets resulted in MMP scores ranging from 0.71 to 0.74, with a maximum predicted model accuracy of 0.70 (0.58–0.81 at 95% confidence interval). In addition, the matrices were highly reproducible between the five HpaII biological replicates (*r*>0.91, *P* value<0.0001 Pearson's correlation coefficient; Supplementary Fig. 1) as well as between HindIII and HpaII data sets (*r*>0.81, *P* value<0.0001 Pearson's correlation coefficient; Supplementary Fig. 2). Even though no significant differences were found between the two enzymes, we decided to use the HpaII interaction matrix (computed as the sum of the five HpaII replicates at a 10 kb resolution, normalized by their respective number of reads) for modelling and subsequent analysis since the HpaII enzyme has a higher cutting frequency.

**Figure 1:**
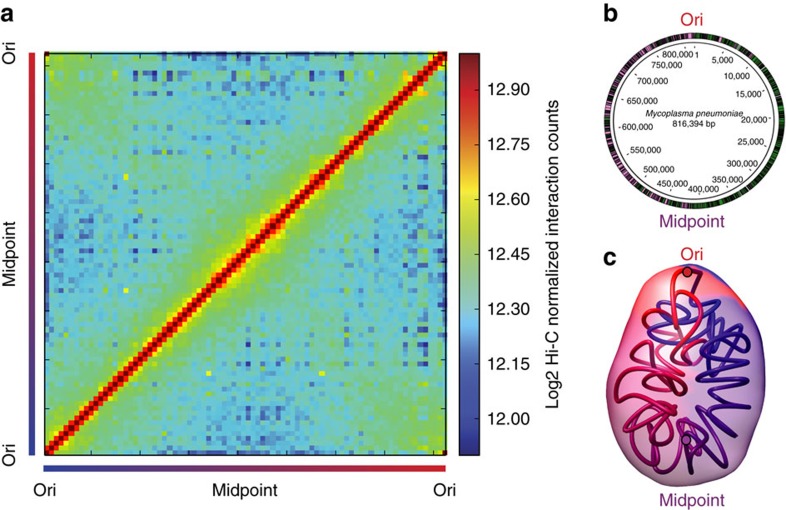
Hi-C matrix and 3D models of the *M. pneumoniae* chromosome reveal a global symmetry with Ori and midpoint located at the two opposite poles. (**a**) Normalized HpaII Hi-C contact map of *M. pneumoniae,* in stationary phase at a 10 kb resolution. The frequency of interactions between a given pair of bins is found at the intersection of the row and column corresponding to those bins. The colour of the contact map, from blue to red, indicates the log2 contact frequency. The bar underneath indicates position along the genome, with Ori being located at a genome coordinate of 0 and midpoint located at ∼ 400 kb. (**b**) Simplified genomic map showing the gene distribution across the chromosome, with black lines delimitating the genes. The colour indicates the strand position, with pink being the − strand and green the + strand. (**c**) A 3D density map representation of the 516 superimposed model structures from the first cluster of *M. pneumoniae* genome models. A central model, referred to as the centroid, is the model that is closest to the mean *x,y,z* coordinates of all the other models. This centroid is shown as a coloured tube starting with particle 1 in blue and ending with particle 82 in red, and uses the same colour code as the bar in **a**. The Ori and midpoint particles are highlighted with red and purple circles, respectively. The lighter colour represents the space occupied by all the models in the cluster, that is, the variability across the cluster.

The resulting Hi-C interaction map has two diagonals intersecting near the centre of the genome, hereafter referred to as the midpoint of the chromosome (at ∼400 kb; Fig. 1a). The location of the Ori was predicted based on the position of the DnaA boxes^46^, and validated by ChIP-seq experiments (Yus *et al*., manuscript in preparation); however, the exact localization of the terminus of replication (Ter) has not yet been experimentally determined in this bacterium. Since in bacteria the Ter is generally located opposite to the Ori, we predicted it to be at this intersection point, and in fact, two putative structural elements having no transcripts (380,176–380,637 bp; 410,901–411,097 bp) were found flanking this region^44^. The main prominent diagonal, characteristic of Hi-C maps, results from the local contact of proximal genomic regions. The second less prominent diagonal (from the upper left to the lower right corner) reflects both the circularity of the genome and the interactions between fragments located on the opposite arm of the chromosome. All together this indicates that the chromosome has a global symmetry extending from the Ori-midpoint axis within the cell, a feature which is further reflected by the distances of genomic bins to the centre of mass (Supplementary Fig. 4). Interestingly, such symmetry is also observed in the linear organization of the genome, where genes are distributed on opposite strands in both the left and the right arms of the chromosome (Fig. 1b).

### Ori and midpoint are located at the two poles

To assess whether the overall symmetry is also reflected in the spatial organization of the genome, we built 3D models of the *M. pneumoniae* genome based on the filtered and normalized 10 kb resolution Hi-C matrix. Briefly, based on the hypothesis that chromatin interaction frequencies are a proxy for spatial proximities between loci^15^, we used TADbit^16^,^47^,^48^ to convert the contact frequencies of genomic loci into spatial distances. Then we searched for the 3D conformations that best satisfied the spatial distances between genomic loci inferred from the frequencies of our Hi-C matrix. A total of 5,000 models were built by an optimization protocol, where the loci were initially placed randomly in 3D space and their positions were modified iteratively using simulated annealing and Monte-Carlo sampling to satisfy as many restraints as possible. Finally, we selected the 1,000 models having the least penalty for not satisfying the imposed restraints and clustered them based on their structural similarity. We found two main clusters corresponding to mirror images of each other, one containing 516 models and the other 484 models. To represent the *M. pneumoniae* chromosome structure model, we created 3D density maps in which the volume occupied by each fragment indicates the variability in the fragment's positioning across the models within the cluster (Fig. 1c). This variability is homogeneous along the entire chromosome. Similar to what was observed for the 3D organization of the *C. crescentus* genome^17^,^18^, our circular chromosome has a global symmetry between the two chromosomal arms that connect the Ori and the midpoint. The Ori and midpoint are located at the two opposite poles of the structure (Fig. 1c and Supplementary Movie 1).

### Validation of chromosome dimensions by DAPI and EM imaging

**Figure 2:**
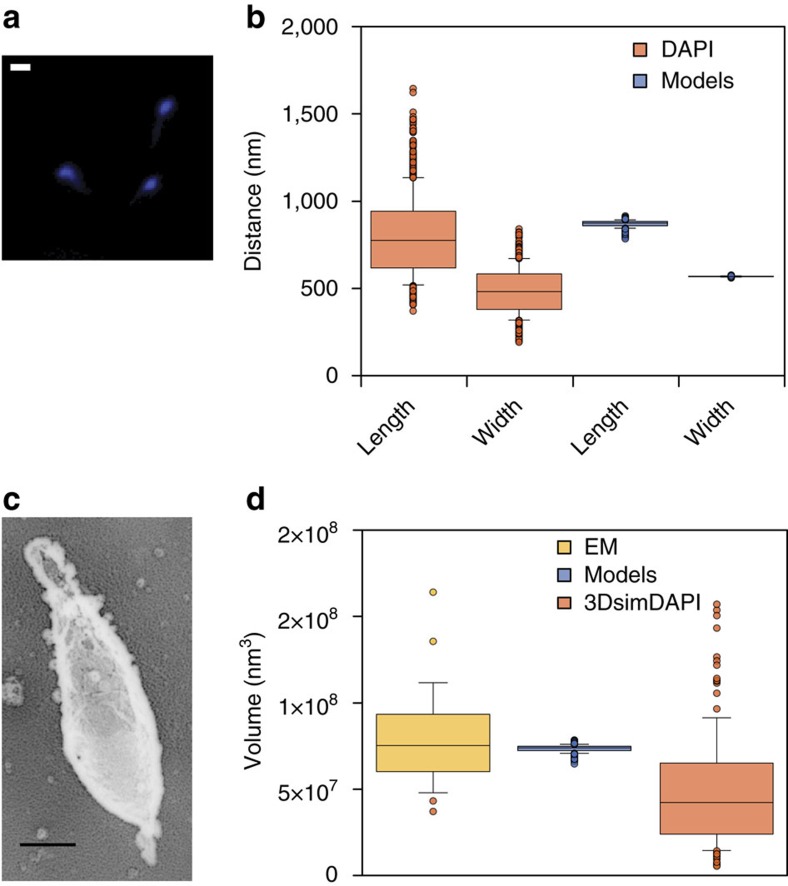
Validation of chromosome dimensions and occupancy by DAPI staining and EM imaging. (**a**) A DAPI-stained image showing the subcellular localization of DNA in *M. pneumoniae*. Scale bar, 1 μm. (**b**) Comparison of the estimated lengths and widths of both the DAPI and the estimated chromosome models in nanometre. Boxplot distribution and median values of length (775 nm) and width (482 nm) over 900 cells, here shown in orange, are estimated from the DAPI images. Boxplot distribution and median values of length (874 nm) and width (568 nm), estimated over 1,000 chromosome models are shown in blue. (**c**) Quick-freeze deep-etch replica TEM imaging of a *M. pneumoniae* cell. Scale bar, 200 nm. (**d**) Distribution and median volume of a *M. pneumoniae* cell based on electron microscopy over 25 cells (0.075 μm^3^; in yellow), on an estimation over 1,000 chromosome models (0.074 μm^3^; in blue), and on DAPI-based three-dimensional super-resolution microscopy over 130 cells (0.042 μm^3^; in orange).

To ensure that the predicted dimensions and volumes of the chromosome models fit within the *M. pneumoniae* cell, we examined cells by transmission electron microscopy (TEM) using the quick-freeze deep-etch replica method^49^ (Fig. 2c). We acquired tilted TEM images of cells and created a 3D reconstruction (Supplementary Movie 2). Although technical limitations did not allow for a 3D reconstruction of the whole cell, we were still able to detect a rotational symmetry along the long cell axis, thereby allowing us to estimate the cell volume. In stationary phase, the median cell volume estimated from the TEM images is 0.075 μm^3^ (standard deviation, s.d. of 0.03 μm^3^).

Also, chromosome dimensions were measured by DNA staining of fixed cells using 4′6-diamidino-2-phenylindole (DAPI) (Fig. 2a). As previously shown, *M. pneumoniae* does not have a defined nucleoid in its centre but rather the chromosome occupies most of the available volume^34^. We measured a median chromosome length of 775 nm (s.d. of 234 nm) and a median width of 482 nm (s.d. of 130 nm). This is comparable to median chromosome length of 874 nm (s.d. of 20 nm) and median width of 568 nm (s.d. of 2 nm) obtained from the 3D models (Fig. 2b). In addition, using a super resolution fluorescence microscopy technique known as 3D-structured illumination microscopy (3D-SIM)^50^, we estimated the 3D chromosome volume by DAPI staining (Supplementary Movie 3), calculating a mean chromosome volume of 0.049 μm^3^ (s.d. of 0.03 μm^3^) (Fig. 2d). The two volumes calculated using microscopy techniques (0.075 and 0.049 μm^3^) are comparable to the chromosome volume estimated from the 3D models (0.074 μm^3^) (Fig. 2d). Altogether, these results support our 3D genome model by indicating that it fits within the biological dimensions of the *M. pneumoniae* cell.

### Validation of 3D models with fluorescence imaging

**Figure 3:**
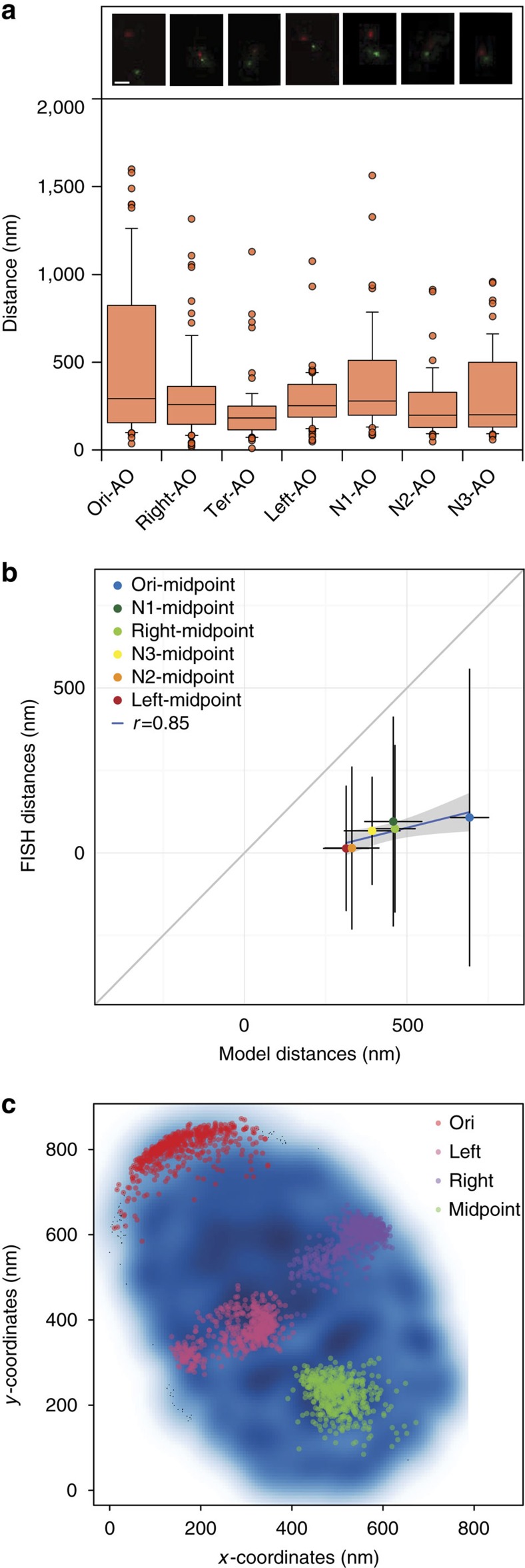
Validation of 3D models with super-resolution imaging. (**a**, top) FISH imaging with red (Alexa Fluor 568) indicating the genomic probes Ori, right, Midpoint, left, N1, N2 and N3, respectively, and green (Alexa Fluor 488) representing the P1 adhesin attachment organelle protein. Scale bar, 200 nm. (bottom) Boxplot distribution and median distances estimated between the genomic probes and AO over ∼50–70 cells. (**b**) Ori-midpoint, N1-midpoint, right-midpoint, N2-midpoint, N3-midpoint and left-midpoint estimated distances from chromosome models in the *x* axis and experimental FISH imaging in the *y* axis. Black lines indicate the variability within the estimated distribution. (**c**) 2D map representation of the chromosomal models from the first cluster shown in blue, with *x* and *y* coordinate positions shown in the *x* axis and *y* axis, respectively. Ori, left, right and midpoint positions across the first cluster of chromosome models are shown in red, pink, purple and green, respectively.

The orientation of the chromosome within the cell body was assessed by combining DAPI staining with immunofluorescence of the P1 adhesin protein localized at the AO^51^. Distances between different genomic regions were determined by super-resolution localization microscopy on non-dividing cells in both the stationary and exponential phase of growth (Fig. 3a,b and Supplementary Fig. 5). We measured distances between fluorescent DNA probes (Fluorescence *in situ* hybridization imaging; FISH) mapped to the Ori (0–1 kb), right (204 kb–205 kb), midpoint (390 kb–391 kb), left (612 kb–613 kb), N1 (99 kb–100 kb), N2 (299 kb–300 kb) and N3 (499 kb–500 kb) loci (Supplementary Table 3), in conjunction with immunofluorescence localization of P1 adhesin (Fig. 3a). The midpoint-AO measurements resulted in the smallest median separation distance of 183 nm, while the right-AO, left-AO, N3-AO and N2-AO distances were larger (258, 252, 200 and 199 nm, respectively). Finally, the Ori-AO and N1-AO distances had the largest separation, with a median distance of 292 and 280 nm, respectively. We repeated some of the measurements on another localization microscope system (Nikon N-STORM) in 3D. As illustrated for the midpoint-AO measurements similar results were obtained (Supplementary Fig. 9). In addition, we calculated approximate distances between midpoint-right, midpoint-left, midpoint-N1, midpoint-N2, midpoint-N3 and midpoint-Ori by deducting the median midpoint-AO distance of 183 nm from their respective distances to the AO (Fig. 3b). The fact that the Ori-AO measurements have a larger variability compared with the others, particularly to the midpoint-AO measurements, suggests that the Ori is not attached to the opposite pole of the cell. The similarity between distances measured in stationary and exponential phases indicates that for non-dividing cells the chromosome structure may not in fact differ (Supplementary Fig. 5).

Next, to assess whether the distances obtained from the 3D models were congruent with the distances obtained experimentally, we computed the Euclidian distances between midpoint-Ori, midpoint-right, midpoint-left, midpoint-N1, midpoint-N2 and midpoint-N3 given their respective coordinates in the 3D models. The midpoint-N2 and midpoint-Ori distances were, respectively, the smaller and larger estimated median distances from both the 3D models (386 and 685 nm) and the imaging data (16 and 109 nm; Fig. 3b). Although the distances estimated from the 3D models are overall larger than the experimental ones, a Pearson correlation of 0.85 is found between the midpoint-left, midpoint-right, midpoint-N1, midpoint-N2, midpoint-N3 and midpoint-Ori median distances estimated from both the 3D models and the fluorescence imaging (Fig. 3b). Thus, overall our imaging data qualitatively validate our 3D models. We conclude that, as was shown in both *B. subtilis* and *C. crescentus*^17^,^52^, the folding of the chromosome is consistent with the linear order of genes along the DNA (Fig. 3c).

### Genes are co-expressed within chromosome interaction domains

**Figure 4:**
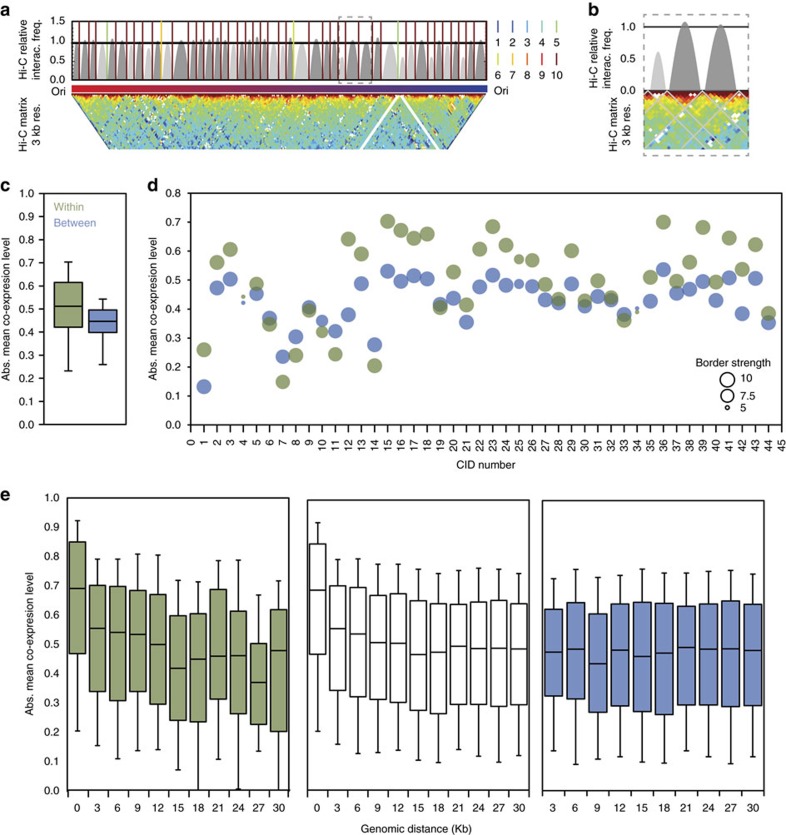
The *M. pneumoniae* chromosome is partitioned into domains of co-expressed genes. (**a**) Hi-C HpaII filtered and normalized contact map at 3 kb resolution, rotated 45° with domain density plots. Each domain is represented by a grey-filled arc and delimited by a coloured line. The height of the domain is proportional to the relative number of interactions in this domain given its size. The colour code from blue to red, numbered 1–10, indicates the border strength or confidence score of the identification of domains. The *y* axis displays the relative Hi-C interaction frequencies and the horizontal line at *y*=1 indicates the expected frequency, given the domain size. If the Hi-C relative interaction frequency inside the CID is higher than 1, that is, higher than expected according to its size, then the domain is coloured in dark grey. Dashed grey rectangle has been zoomed-in in b. (**b**) Zoom-in region from **a** of three consecutive domains. Domain border are represented by grey lines in the Hi-C matrix. (**c**) Absolute mean co-expression distribution of gene pairs, when both genes are located within the same domain as shown in green, or genes between two different domains as shown in blue. Co-expression refers to the degree by which genes change in the same direction under different perturbations, between all pairs (i,j) of genes. Here, we compared the absolute mean co-expression of pairs of genes within and between domains. (**d**) Detailed absolute mean co-expression distribution across the 44 domains. Point sizes are proportional to border strength. The colour depicts, as before, the two cases of gene pairs within the same domain, shown in green, and gene pairs between different domains, shown in blue. (**e**) Absolute mean co-expression distribution as a function of genomic distance, with distances between gene pairs smaller than 30 kb for the same two cases as in b and any gene pairs across the whole genome, as shown in white.

In Hi-C interaction maps, a significant proportion of the signal lies in the vicinity of the diagonal where most of the interactions occur. We have used this property to further increase the resolution of our maps to 3 kb bins. Although such maps have low scores for accurate 3D modelling (the MMP score of the maps at 3 kb was 0.56; Supplementary Fig. 3), they can be used for studying the local organization of chromatin, omitting long-range interaction data. We used the TADbit program^16^,^47^,^48^ to segment the HpaII matrix (at 3 kb resolution) into 44 CIDs (Fig. 4a,b). Moreover, TADbit assigned a confidence score to each domain border ranging from 1 to 10, the higher the score, the higher the confidence (Fig. 4a). The CID sizes ranged from 15 to 33 kb, with a median size of 15 kb. This is smaller than those previously found in *C. crescentus*, which range from 30 to 420 kb (ref. 18). Moreover, we performed the alignment of domain borders across five HpaII replicates and confirmed that domain borders are conserved across replicates (*P* value<0.0001 randomized domains boundaries alignment test; Supplementary Fig. 7). To ensure that the identified domain borders were not artefacts arising from the location of restriction sites, we calculated the number of HpaII sites present in borders. The analysis confirmed that the domain borders were not significantly enriched with HpaII sites (permutation test *P* value=0.868). We also looked at other properties such as gene localization, Clusters of Orthologous Groups function, TFs enrichment, termination of genes, and methylation levels, but none were found to be significantly enriched with the permutation test. Interestingly, GC content was found to change significantly, with a lower percentage value being found at the domain borders (permutation test *P* value=0.088). In addition, a significant number of both convergent (genes pointing towards each other in the direction of transcription in the two strands) and divergent gene pairs (genes pointing in opposite directions of transcription in the two strands) were found at these borders (permutation tests *P* value=0.026, *P* value=0.037, respectively).

To assess whether the local organization of the *M. pneumoniae* genome into CIDs is related to transcriptional regulation, we compared the absolute mean co-expression of pairs of genes within and between domains. Interestingly, we found that genes are significantly co-expressed within domains (*t*-test *P* value=0.0032; Fig. 4c). Specifically, higher mean absolute co-expression values were observed for genes in 34 out of the 44 domains (Fig. 4d). A border permutation test suggested that a low co-expression is found at the border of these domains (*P* value=0.11 permutation *t*-test). Moreover, these results also indicate that the higher co-expression levels of genes within CIDs are not only due to genomic linear proximity (Fig. 4e). Indeed, independent of the CID in which the genes are located, proximal genes have higher co-expression than distant genes^53^. However, the co-expression is stronger for proximal genes (<12 kb apart) when they are located within the same CID, while it is weaker for any proximal genes across the whole genome (*t*-test *P* value<0.0001; Fig. 4e). Interestingly, these correlation trends are not observed when the genes are located in different CIDs, that is, separated by a CID border.

The *M. pneumoniae* transcriptome comprises 671 operons, being mostly monocistronic or bicistronic, and 852 suboperons with a median size of 605 bp and a maximum size of 9,197 bp. In comparison, the domains identified in our study are larger, having a median size of 15 kbp. As an illustration of the distribution of operons across domains, we plotted the 20 and 14 suboperons found in CID 8 and 9, respectively (Supplementary Fig. 8). The average number of operons and suboperons found within a domain is 15 and 18, respectively. Our analysis shows that these different operons of the same domain tend to be more co-regulated than operons between different domains. Altogether, our results demonstrate that the mean co-expression level observed for genes of the same domain is higher than the mean co-expression level observed for genes between domains, revealing that the genome is partitioned into domains of co-expressed genes.

### Inhibiting supercoiling reduces domain borders sharpness

**Figure 5:**
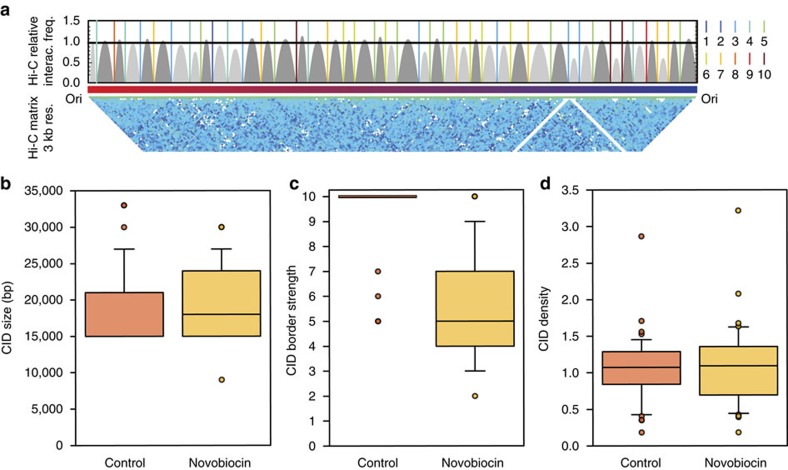
Inhibiting supercoiling decreases the sharpness of domain borders. (**a**) Same as Fig. 4a, but with Hi-C HpaII Novobiocin-treated contact map at 3 kb resolution. (**b**–**d**) Boxplot distributions and median values of CID size, border strength and density distribution in wild-type (orange) and Novobiocin-treated (yellow) cells. The CID density is computed as the sum of all the Hi-C interactions in a domain divided by the expected number of interactions, where the expected number of interactions is computed as an average for each genomic distance^71^. The units of the CID density are thus interactions normalized by the genomic distance.

To study the effect of inhibiting supercoiling on chromosome structure, we performed Hi-C on cells treated with novobiocin, a drug that inhibits DNA gyrase and DNA negative supercoiling. The outcome of DNA gyrase inhibition with novobiocin is the relaxation of the DNA^54^. The novobiocin-treated cells resulted in Hi-C interaction maps with 42 CIDs (Fig. 5a), ranging from 9 to 30 kb and with a median size of 18 kb (Fig. 5b). When comparing CIDs in novobiocin-treated cells to the control, we found that 15 CIDs were conserved. Interestingly, the TADbit median confidence score for the domain borders was five out of 10 compared with 10 for non-treated cells (Fig. 5c). However, the domain densities, that is the relative number of interactions given the domain size (computed as the sum of Hi-C interactions in a domain divided by their expected interactions), were not significantly altered in novobiocin-treated cells (Fig. 5d). Taken together, and as reported in *C. crescentus*^18^, novobiocin reduces the sharpness of CID borders, thereby suggesting that supercoiling may regulate domain formation in bacteria.

## Discussion

Chromosome conformation capture based experiments coupled with deep sequencing have been used to explore bacterial genome organization and its role in transcriptional regulation^17^,^18^,^20^,^21^. Here, we have analysed the 3D genome organization of the *M. pneumoniae* bacterium, a model organism with a small genome size and a simplified gene regulatory network. Compared with other bacteria, *M. pneumoniae* not only has considerably less TFs and NAPs, but also the total copy number of these proteins is proportionally lower. More specifically, in *M. pneumoniae* there are only the SMC and histone-like IHF NAP proteins and two sigma factors responsible for coordinating gene transcription. By combining Hi-C and super-resolution fluorescence imaging, we were able to identify fundamental principles of genome organization, such as the partitioning of a reduced genome into domains. Furthermore, we provide evidences that genes inside CIDs tend to be co-regulated, indicating that the chromosome structure has a role in transcription regulation.

The *M. pneumoniae* genome contact map revealed a double diagonal intersecting near the centre of the genome. This corresponds to the midpoint and reflects a global symmetry of the genome along the Ori-midpoint axis. The 3D models generated of the genome conformation resulted in the Ori and midpoint loci being located at the two opposite poles of the structure. In addition, our DAPI staining and TEM images indicated that the 3D chromosome models fit within the dimensions of the *M. pneumoniae* cell. It is important to take into consideration that the limited resolution of DAPI imaging impeded an accurate estimation of the nucleoid occupancy.

Using super-resolution fluorescence imaging, we corroborated our 3D models of chromosome conformation. The distances estimated from the 3D models obtained using Hi-C data were overall larger than the ones obtained from super-resolution imaging of cells. This can be explained by the limited resolution of our models (10 kb), as the actual occupancy of a bin within the cell has not been determined. Moreover, the fixation and permeabilization treatments of cells for FISH can induce shrinkage artefacts. Imaging indicates that the midpoint locus is the closest of all tested loci to the AO in both stationary and exponential phases. In *M. pneumoniae*, the duplication of the AO was reported to be coordinated with cell division, which is known to occur by binary fission^55^. Furthermore, during cell replication but before nucleoid separation, the migration of the AO to the opposite pole of the cell has been observed in fixed cells, suggesting a coordination between the AO duplication and DNA replication^34^. Once a new organelle has formed, it remains attached to the surface while the old AO pulls the dividing cell away from the nascent organelle, positioning itself at the opposite pole^56^. Similarly, as described for the closely related species *Mycoplasma gallisepticum*^57^, our findings suggest a possible anchoring of the DNA to the AO near the midpoint. Unfortunately, the observed cell-to-cell variability in the Ori-AO and midpoint-AO distances did not allow us to demonstrate that the AO is attached to a specific chromosome region as division occurs. Technical restraints of the FISH protocol only allowed the study of fixed cells, thereby limiting a deeper understanding of cell division in *M. pneumoniae*.

**Figure 6:**
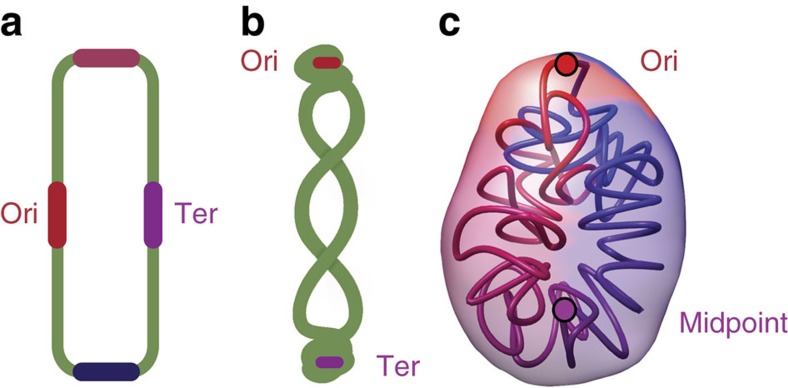
Models of bacterial chromosome organization. Models of nucleoid organization with Ori and Ter represented by red and purple circles. (**a**) Model of the *E. coli* genome with the four macro-domains Ori, Ter, left, right, represented by circles in red, purple, pink and blue, respectively. (**b**) Model of the *B. subtilis* genome adapted from ref. 52. (**c**) 3D models of the *M. pneumoniae* genome conformation.

Previously, a double diagonal was also observed in the contact map of the phylogenetically, closely related gram-positive bacterium *B. subtilis*^21^,^22^,^23^, as well as in two gram-negative bacteria *C. crescentus*^17^,^18^ and *Vibrio cholera*^21^. Interestingly, this symmetry observed along the two replichores was not observed in *E. coli,* which has an open chromosome structure (Fig. 6a)^20^,^21^, likely due to the orientation of the chromosome within the cell. Indeed, in *B. subtilis* and *C. crescentus,* the Ori and Ter are preferentially located at opposite poles early on in the cell cycle^17^,^52^, and both have an origin proximal region parS (partition system) that assists in the orientation of the chromosome during replication^58^,^59^. In the case of *C. crescentus*^17^ the Ter is situated at the new pole and the left and right extend along the cell^17^ (Fig. 6b), whereas in *E. coli* the left and right are situated towards the two poles and the Ori and Ter are closer to the middle of the cell^60^ (Fig. 6a). The chromosome organization of *C. crescentus* is similar to that of *M. pneumoniae* in the fact that the Ori and midpoint (or Ter in *C. crescentus*) are localized at the two opposite poles (Fig. 6c), but differ in the fact that the chromosome of *C. crescentus* has an ellipsoidal form with periodically arranged arms twisting around each other^17^.

Several published studies have previously shown that mammalian genomes are partitioned into TADs^24^,^25^, which range from 200 kb to 1 Mb and are conserved across different species and cell types. Our analysis allowed the detection of bacterial TAD-like domains (CIDs) for the first time in *M. pneumoniae*. More specifically, *M. pneumoniae* is partitioned into a total of 44 CIDs, which range from 15 to 33 kb in size and are smaller than those reported for *C. crescentus*^18^ and *B. subtilis*^22^,^23^. These *M. pneumoniae* CIDs could be related to the 10 kb loops that are bounded by stochastic barriers as suggested for *E. coli*^6^,^7^ and *Salmonella enterica*^61^. In contrast, the nested domains reported for *C. crescentus*^18^ and *B. subtilis*^22^,^23^ may be more related to the macrodomain-like regions identified in *E. coli*. These findings suggest that domains are an elemental component of genome organization throughout the evolution of all organisms. Future work using super-resolution imaging of CIDs could estimate how variable the CIDs in prokaryotic cells are compared with the previous results of TADs in eukaryotes^62^. Furthermore, it has not been shown that a specific chromosome structure is essential for genomic functions as large genome reduction done in *Mycoplasma mycoides*^63^ or genome reorientation^17^ does not seem to compromise cell viability. Thus having a defined chromosome structure seems to be important, but it can be altered. However, large genome DNA rearrangements can have important effects on the presence, order and expression of genes^64^, suggesting that chromosome domains are a fundamental principle of genome folding.

We also observed, as previously reported for *C. crescentus*^18^, that inhibition of supercoiling by novobiocin significantly reduced the sharpness of CIDs. Our finding suggests that supercoiling could be regulating domain formation in bacteria. Interestingly, our analysis also indicates that genes inside CIDs tend to be co-regulated, with lower co-expression levels being detected between genes at the domain boundaries. In *C. crescentus,* it was previously reported that domain borders correlated with the presence of highly expressed genes, being as the DNA is kept free of plectonemic loops during active transcription^18^. Although we did not observe such a correlation in *M. pneumoniae*, we established that not only domain borders are correlated with the presence of convergent and divergent gene pairs but also that borders are characterized by low GC content levels. Promoter orientation has been related to gene expression regulation and more particularly divergent transcription could create negatively supercoiled or underwound DNA, while AT content has been previously related to the physical properties of DNA such as DNA curvature. Similarly, it has also been reported in *E. coli* and *Salmonella typhimurium* that the localization of domain loop boundaries is found in AT-rich regions^65^, and more recently in *B. subtilis* that a considerable number of domains barriers overlap with AT-rich sequences acquired through horizontal gene transfer^22^. Even though the contribution of NAP and SMC proteins in the global genome organization was recently refuted in *E. coli*^20^, as well as their role in the formation of CIDs in *C. crescentus*^18^, the formation of these domains was consistent with the distribution of histone-like proteins binding sites, such as H-NS, HU, Fis and IHF. As *M. pneumoniae* however has a limited number of copies of the histone-like IHF protein^41^, thereby making it difficult to maintain the CID boundaries, it is likely that additional factors contribute to the formation of such domain loops. Furthermore, since *M. pneumoniae* only has a handful of DNA-binding proteins and very few TFs (Table 1), it is intriguing that it is capable of establishing a well-defined chromosome structure as well as maintaining CID boundaries. We speculate that very few factors (such as the SMC protein) are necessary to define a 3D chromosome structure and provide evidence that other elements like supercoiling could regulate these domain boundaries, which are characterized by low GC-content and might be related to the physicochemical properties of DNA.

## Methods

### Overview of methodology

With the aim of reconstructing the 3D chromosome of *M. pneumoniae* we first performed Hi-C experiments, which enabled the purification of ligation products and subsequent massive parallel sequencing^15^. Next, all fragments of reads were mapped to the *M. pneumoniae* genome with the iterative mapping method ICE^45^, and then further filtered and normalized^45^ to obtain a genome-wide chromatin contact map. Next, the MMP score of the matrix^47^ was computed to assess its modelling potential. Finally, 3D models of the *M. pneumoniae* genome were generated using TADbit^16^,^48^. To validate the computed 3D genome architecture, fluorescence and electron microscopy were performed to estimate the cell dimensions and cell volume, as well as the distances between different chromosomal regions.

*Hi-C protocol with a 6-cutter*. To fix the long-range DNA interactions^15^, 3 × 10^9^ *M. pneumoniae* M129 cells were grown in 150 cm^2^ flasks for 6 h (exponential phase) or for 96 h (stationary phase), and cross-linked with 1% formaldehyde (methanol free, Pierce) for 10 min at room temperature (RT). The reaction was stopped with 0.125 M glycine and cells were washed before lysis. Four millilitre of lysis buffer (10 mM Tris·HCl pH 8.0, 10 mM NaCl, 0.2% NP-40, protease inhibitors from Roche, 1 mM EGTA) was added and cells were broken with the help of a syringe/G25 needle (5 × ). The lysate was distributed into four tubes and spun in a tabletop centrifuge at 2,500*g* for 5 min. The supernatant was removed and three pellets frozen for later use. One chromatin pellet was washed twice with 1.4 ml of NEBuffer 2/3 (HindIII). After resuspension in 1 ml of NEBuffer 2/3, 10 μl of 10% SDS was added, mixed carefully and incubated at 65 °C for 10 min to allow accessibility of restriction enzymes. Tubes were placed back on ice immediately after incubation. SDS was quenched by adding 110 μl of 20% Triton X-100 and mixed carefully. Chromatin was digested by adding 100 μl of 20,000 U ml^−1^ HindIII +5 mM EGTA and incubated at 37 °C overnight (O/N) while shaking. The next steps included marking the DNA ends with biotin and performing blunt-end ligation of crosslinked fragments. This last step allows ligation junctions to be posteriorly purified. To fill in the restriction fragment overhangs and mark the DNA ends with biotin, 5 μl of a mixture containing 10 mM dATP, dGTP and dTTP, 62.5 μl 0.4 mM biotin-14-dCTP, and 41 μl 2 U μl^−1^ Klenow was added to the Hi-C tubes, mixed carefully and incubated for 45 min at 37 °C. To inactivate the enzymes, 250 μl of 10% SDS was added to the Hi-C tubes, before incubation at 65 °C for exactly 30 min, placing on ice immediately afterwards. The ligation was performed under extremely dilute conditions in order to favour ligation events between the cross-linked fragments. Working on ice, 9 ml ligation mix (0.5 ml 20% Triton X-100, 1 ml 10x T4 ligation buffer, and 7.5 ml water) was added to a 50 ml falcon tube and the digested chromatin was incorporated into the mixture of the corresponding tube. After mixing by inverting the tubes, the ligation was performed for 4 h at 17 °C. Crosslinks were reversed and proteins degraded by adding 50 μl of 20 mg ml^−1^ proteinase K per Hi-C tube and incubating the tubes O/N at 65 °C. An additional 50 μl of 20 mg ml^−1^ proteinase K was added per tube the following day and incubated at 65 °C for another 2 h. The reaction mixture was cooled to RT and DNA was purified by performing an extraction in Maxtract tubes (Qiagen) with one volume of phenol pH 8.0, and then with phenol/chloroform/IAA (25:25:1) (at each step the tube was vortexed for 2 min, spun for 5 min, 1,500*g*, at RT and as much as possible of the aqueous phase was carefully transferred to a new 50 ml tube). Then DNA was precipitated by adding 2 μl glycogen, 0.1 × volumes of 3 M sodium acetate, pH 5.5 and 2 × volumes ethanol, left 30 min at −20 °C and spun 25 min at 12,000*g* (Beckman-Coulter 25,50 rotor) at 4 °C. The pellet was washed with ∼5 ml 75% ethanol and air-dried before dissolving it in 400 μl TE (10 mM Tris·HCl pH 8.0, 1 mM EDTA). The DNA mixture was transferred to a clean 1.5 ml centrifuge tube. Another round of purification was performed by doing one phenol/chloroform/IAA extraction and DNA precipitated by adding 0.1 × volumes of 3 M sodium acetate, 2 × volumes of ethanol and incubating 30 min at −80 °C. After spinning down the precipitated DNA, the DNA pellet was washed with 70% ethanol and resuspended in 25 μl TE. To degrade any RNA that might be present, 1 μl of 1 mg ml^−1^ RNAse A was added per tube and incubated for 30 min at 37 °C. As some fragments do not get ligated, the exonuclease activity of T4 DNA polymerase was used to remove the biotin, thereby avoiding any unligated fragments getting pulled down later. This was done by mixing ∼5 μg (∼25 μl) of Hi-C DNA with 1 μl of 10 mg ml^−1^ BSA, 10 μl of 10 × NEBuffer 2, 1 μl of 10 mM dATP, 1 μl of 10 mM dGTP and 5 U of T4 DNA polymerase in a total volume of 100 μl and incubated at 12 °C for 2 h. The reaction was stopped by adding 2 μl of 0.5 M EDTA pH 8.0. To purify the DNA, a phenol/chloroform/IAA extraction followed by ethanol precipitation was done as described above. The supernatant was discarded and the DNA pellets resuspended in 50 μl of water. Then the DNA was sheared and size selected, to obtain a uniform size suitable for high-throughput sequencing. The DNA must be sheared to a size of 300–500 bp with a Covaris nebulizer (10% duty cycle, intensity: 2,200 cycles, 45 s at 4 °C) in a minimum of 55 μl of TE. The concentration was measured with Qubit (DNA, High sensitivity, Invitrogen). To repair the sheared DNA ends, the Next (NEB) protocol was followed (blunting and A tailing). Subsequently the junctions were enriched by biotin pull-down, thus allowing for the identification of interacting chromatin fragments by paired-end sequencing, as follows: Ligation junctions were purified from the DNA pool, first, 150 μl of resuspended streptavidin Dynabeads (Invitrogen) beads were washed twice with 400 μl of Tween Buffer (TB: 5 mM Tris·HCl pH 8.0, 0.5 mM EDTA, 1 M NaCl, 0.05% Tween). All washes were done in the same manner: (i) buffer added to the beads; (ii) sample rotated for 3 min at RT; (iii) sample spun briefly to collect all of the suspension; (iv) beads recovered using a magnetic particle concentrator; and (v) supernatant removed and beads resuspended in 600 μl of No Tween Buffer (NTB: 5 mM Tris-HCl pH 8.0, 0.5 mM EDTA, 1 M NaCl) plus Hi-C DNA (∼500 ng). Binding was allowed by incubating the mixture at RT for 15 min with rotation, and recovering the DNA-bound streptavidin beads as above, before washing in 400 μl of NTB followed by 100 μl of T4 ligase buffer (NEB). Finally the beads were resuspended in 50 μl of ligation buffer and Illumina paired end adapters were ligated (ratio: 1 μl of 2 μM primers per 10 ng of DNA) with 1,200 Units of T4 DNA Ligase (NEB) for 2 h at RT. Non-ligated adapters were removed by recovering the Hi-C DNA bound beads and washing them twice with 400 μl of TB, once with 200 μl of NTB, and finally once with 200 μl and then 50 μl of NEBuffer 2. After the last wash, the beads were resuspended in 25 μl of NEBuffer 2. The library was PCR amplified with Phusion (Next kit, NEB): 2 μl of the suspension in a 50 μl reaction, and 1.0 and 2.1 Illumina primers (1 μl, 10 uM), for 16 cycles and sequenced in the HiSeq Illumina platform.

*Hi-C protocol with a 4-cutter*. Chromatin was prepared as above^20^. When indicated, 100 μg ml^−**1**^ of novobiocin (Sigma) was added directly to the medium 30 min before fixation. Cells were lysed with 4 ml of Hi-C Lysis buffer (10 mM Tris·HCl pH 8.0, 10 mM NaCl, 0.2% NP-40, 5 mM EGTA and protease inhibitors) at 4 °C, passed 5 × through a syringe/G25 needle and chromatin collected by centrifugation (2,500*g* for 5 min, 4 °C, tabletop centrifuge). Only one pellet was used (the rest were frozen at −80 °C), and was washed twice in 1 ml of NEBuffer 1 plus 5 mM EGTA at 4 °C. Before digestion, chromatin was solubilized by adding 300 μl of NEBuffer 1, 5 mM EGTA and 0.1% SDS, incubated for 1 h at 37 °C, and stopped with TX-100 (2% final). Afterwards, 100 U of HpaII was added and incubated O/N at 37 °C. The reaction was stopped adding SDS to a final concentration of 1.3% and incubated for 1 h at 50 °C. Half of the sample was ligated by adding 5 ml of 10 × NEB T4 Ligase buffer, 2.5 ml of 20% Tx-100, 0.5 ml of 0.5 M EGTA, in a final volume of 50 ml, and ligated with 20 U (50 μl) of T4 DNA ligase (NEB), O/N at 16 °C. To de-crosslink the sample, 375 μl of 20 mg ml^−1^ proteinase K was added for 2 h at 65 °C and purified by phenol extraction, Maxtract resin, and ethanol precipitated as above. Further fragmentation was performed with Covaris to reduce the size of the DNA to ∼200–500 bp (Duty cycle: 10%, int.: 2,200 cycles, 20 s at 4 °C). DNA was submitted to the CRG Ultrasequencing facility for standard Illumina library preparation and paired-end sequencing.

*Genomic DNA preparation*. For the controls without formaldehyde fixation, genomic DNA was prepared as in (Yus *et al*.)^40^ and digested and religated as above (without the need for de-crosslinking). The same equivalent concentration was used in order to keep the infinite dilution conditions.

### Generation of contact matrices

To construct the interaction maps of the *M. pneumoniae* genome, read pairs of 50 bp were uniquely mapped to the MPN129 reference genome (NC_000912, NCBI) covering 816,394 bp, using Bowtie2, and following the iterative mapping strategy ICE from the hiclib Python library^45^. The optimal start and end positions for mapping were determined using the fastq quality of the read, and set to 4 and 44, respectively. The minimal size for mapping was set to 25 bp. The iterative mapping procedure starts with a read length of 25 bp and increases by steps of 3 bp until a maximal read length of 40 bp is reached. Only read pairs for which both reads uniquely aligned to the genome were considered in subsequent steps. The MPN129 genome was divided into restriction fragments (449 HindIII fragments, or 1,411 HpaII fragments) and each read of a read pair was sorted into its corresponding restriction fragment. Read pairs were classified as valid Hi-C products, non-ligation products, or self-ligation products, and only the valid Hi-C products were subsequently considered below. We then constructed a genome wide matrix, M, of different resolutions (3, 5, 10, 15 and 20 kb) by dividing the genome into 3, 5, 10, 15 and 20 kb bins, and pooling interactions into their corresponding bins. To correct for possible Hi-C artefacts, the matrix was then filtered and normalized using the methodology of iterative correction from the hiclib Python library^45^ as done in a previous study for *C. crescentus*^18^. Essentially, the number of interactions, or read counts was converted into Hi-C scores by applying the following equation and iteratively repeating it for the resulting contact map after each cycle: mij=mij * (total reads)/(total reads in bin i * total reads in bin j). The iterative procedure was repeated until the maximum relative error of the total number of Hi-C scores in a bin was less than 10^−5^. The total number of reads before and after filtering are shown in Supplementary Table 2. In addition, using a control library without formaldehyde fixation, computed as the sum of three control replicates (*r*>0.6, *P* value<0.0001 Pearsons' correlation test), we filtered interactions off-diagonal and off-diagonal plus one, which are not due to 3D contacts in the chromosome, representing ∼2.6% of the total number of cells in the matrix. These interactions were found in two regions with a high sequence similitude computed by the Needleman-Wunsch global sequence alignment with EMBOSS Needle^66^, which justifies possible PCR artefact amplification for repetitive sequences. The affected bins were: 2, 5, 7, 11, 12, 13, 14, 15, 17, 18, 19, 20, 21, 25, 34, 35, 42, 44, 45, 50, 57, 58, 61, 62, 71, 74, 78. Subsequent analysis and visualization was done using R scripts.

### Reproducibility of Hi-C data

To analyse the reproducibility between all the HindIII and HpaII replicates in stationary phase, and between HpaII in exponential and stationary phase, we decomposed the two-dimensional matrices of normalized and filtered data sets into two, one-dimensional vectors row-by-row and computed the Pearson correlation coefficient between the two vectors with R (Supplementary Methods).

### Matrix modelling potential using MMP score

We computed the MMP score of the matrix to assess its potential for modelling, and also computed the predicted accuracy of the models, named distance Spearman correlation coefficient, using the MMP score python script^47^. MMP score is based on the matrix size, the contribution of significant eigenvectors in the matrix and the skewness and kurtosis of the *z*-scores distribution of the matrix.

### Integrative 3D modelling with TADbit

The HpaII Hi-C matrix was used for modelling at a resolution of 10 kb after filtering by hiclib methodology^45^ and additional filtering using a control library as previously mentioned. To build the 3D models, we applied a restraint-based modelling approach using the TADbit python library^16^,^47^,^48^. The genome was defined by 82 particles, determined by the resolution of the contact map at 10 kb. Each particle had a radius of 100.5 nm that was determined empirically with the scale parameter of 0.0201, nm per bp. First, TADbit identified empirically three optimal parameters using a grid search: (i) the proximal distance between two non-interacting particles set as 250 nm; (ii) a lower-bound cutoff to define particles that do not interact frequently, set as −0.6; and (iii) an upper-bound cutoff to define particles that do interact frequently, set as −0.2. Subsequently, considering an inverse relationship between the frequencies of interactions of the contact map and the corresponding spatial distances, TADbit translated the frequencies of interactions into spatial restraints between particles. Two consecutive particles were spatially restrained at an equilibrium distance that corresponds to the sum of their radii. Non-consecutive particles with contact frequencies above the upper-bound cutoff were restrained at an equilibrium distance, while those below the lower-bound cutoff were maintained further than an equilibrium distance. Second, TADbit used a Monte Carlo simulated annealing sampling procedure to identify 3D models that best satisfy all of the imposed restraints. The contact map obtained from the final models resulted in a Pearson correlation of 0.83 with the input Hi-C interaction matrix, which is indicative of good model accuracy^47^.

### 3D reconstruction of TEM imaging and cell volume

Mycoplasma cells were scraped into fresh growth medium at 50-fold the concentration of the original culture. The cell suspension was put on a 3 × 3 mm piece of glass and left at 37 °C for 15 min. The cells on the glass were fixed with 1% glutaraldehyde in PBS containing 75 mM sodium phosphate (pH 7.3) and 68 mM NaCl for 3 min at RT, rinsed with 0.2 ml PBS once and then thoroughly washed in water. The fixed cells were frozen at a liquid nitrogen temperature using CryoPress (Valiant Instruments, St Louis, MO), deep-etched, rotary-shadowed by platinum at an angle of 30°, and backed with carbon in a jet freezing device vacuum (JFDV) freeze-etching machine (JEOL Ltd, Akishima, Japan). Replicas were floated from the glass by slowly immersing the surface into full-strength hydrofluoric acid, cleaned with commercial bleach, rinsed in water, and picked up onto Formvar-coated 400-mesh copper grids as described^67^. Series of replica images were taken by tilting the sample stage over 30° to both sides in 5° intervals, using a transmission electron microscope (JEM1010, JEOL) at 80 kV.

TEM images tilted with angles −30, −25, −20, −15, −10, 0, 5, 10, 15, 20, 25, 30 were registered by cross-correlation with Matlab and rotated to ensure a vertical rotation axis. The outline of the cell in each image was determined by thresholding using Fiji, followed by manual removal of background contamination and filling of gaps inside the bacteria. From these binarized images the sample area could be extracted by counting pixels within the cell area. The volume of the cell was then calculated by assuming each cell is rotationally symmetric along its long axis (Supplementary Movie 3). The cell was segmented into cylinders and cones along this axis, and the volume was computed as the sum of both the cylinder and cone volumes as follows:


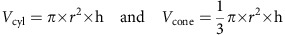


where *V*_cyl_ is the volume of a cylinder of height *h* and radius *r*, and *V*_cone_ is the volume of a cone of height *h* and radius *r*.

To reduce the error caused by inaccuracies in manual image editing and/or a lack of rotational symmetry, the final volume was calculated as the average of the volumes determined for individual images.

### DAPI imaging and measurement of DNA content

*M. pneumoniae* cells were grown in a 75 cm^2^ flask in Hayflick medium for 4 days under standard conditions. After 4 days, the medium was removed and cells were re-suspended in 5 ml of fresh Hayflick medium, and subsequently scrapped and collected. Aggregates were then removed by passing the cells through a syringe with a G25 needle (10 × ) and a 0.45 μm filter, and mixed with 5 ml of 6% gelatin. Then, cells were grown on borosilicate coverglass slides (Thermo Scientific) for 6 h. Cells were fixed in a final concentration of 4% formaldehyde (Pierce) for 20 min at RT followed by 40 min at 4 °C, before further fixing with cold methanol at −20 °C O/N. DAPI was added on the slides (10 μg ml^−**1**^) for 15 min and three washes were done with PBS.

The cells were observed on Axio Observer Z.1 equipped with a × 63 1.4 Oil objective and an Axio-Cam MRm camera. The fluorescence intensities of DAPI were measured by using the command ‘analyse particles' using Fiji and taken as measurements of the DNA contents of individual cells.

### 3D-SIM imaging and chromosome volume

*M. pneumoniae* (M129 strain) was cultured in modified Hayflick's medium at 37 °C till confluency in a T-25 tissue culture flask. *M. pneumoniae* cells were scraped in 10 ml and clumps were broken by a 26 gauge syringe. A dilution of 1 in 100 of *M. pneumoniae* cells in Hayflick's medium was grown overnight on #1.5 coverslips (Proscitech) at 37 °C. After three washes with PBS, cells were fixed in 2% paraformaldehyde in PBS for 60 min. Coverslips were washed again with PBS and paraformaldehyde was quenched with 100 mM glycine in PBS for 5 min. Cells were permeabilised with 0.5% Triton X-100 for 5 min followed by three washes with PBS. To visualise the chromosome, 0.4 μg ml^−**1**^ DAPI (Roche) was added to cells for 5 min. Coverslips were washed with PBS, dried and mounted with VECTASHIELD (Vector Labs) onto a glass slide for imaging.

3D-SIM was performed with a V3 DeltaVision OMX 3D-SIM Blaze system (GE Healthcare, Issaquah, USA). Solid-state lasers provided wide-field illumination and interference patterns for 3D-SIM were generated by interfering light beams^68^ The images were captured using a 60 × 1.4 NA UPlanSApo oil objective (Olympus, Japan) and scientific complementary metal oxide semiconductor (CMOS) 512 × 512 pixel 15-bit cameras (pco.edge, PCO AG, Kelheim, Germany), and were sectioned using a 125 nm Z-step size. Raw 3-phase images were reconstructed as previously described^50^.

Reconstructed images stacks were 3D rendered and presented using IMARIS software (v8.1, Bitplane AG). First the image was 3D cropped to remove the top layer due to fluorescence reflection. DAPI channel contrast was adjusted to increase intensity of single-cell fluorescence (linear adjustments only). A surface layer (DAPI channel) was added with the following parameters: ‘Surfaces Area Detail Level' set to 0.8 μm and ‘Thresholding: Background Subtraction (Local Contrast)' set to 0.3 μm. The 'Threshold: Threshold (Background Subtraction)' was adjusted until the surface overlay aligned with the fluorescence of the DAPI. The ‘Number of Voxels' above 10.0' filter was added to generate the surface layers. Volumes in μm^3^ was then extracted as CSV files.

### Median dimensions and volume of the chromosome models

The median length, mean width and mean volume of the chromosome were computed by taking the median of the lengths, widths and volumes of the 1,000 models. The length of the chromosome in each model was estimated as the distance between the two most distant particles and the width as the double of the radius of gyration of the model. To calculate the volume of each model, we computed the volume of a prolate ellipsoid, using the estimated length of the chromosome as the major axis and the width of the chromosome as the diameter of the minor axis.

### FISH combined with immunofluorescence

We estimated the distances between four regions of interest corresponding to the four quarters of the circular *M. pneumoniae* genome, namely Ori, right, midpoint and left as shown in Supplementary Table 3. This was done by combining FISH with immunofluorescence of the P1 protein localized at the AO^51^.

The resolution of a regular fluorescence microscope image is limited by diffraction to about half the wavelength of the emitted light, which due to the small size of *M. pneumoniae* does not allow localizing a region marked with a fluorescent probe. To overcome the diffraction limit, we used a high-resolution microscope with ground-state depletion (GSD) followed by individual molecule return (GSDIM), which improves resolution down to 20 nm (refs 9, 10, 11, 12). The principle of GSDIM relies on ensuring that only a few illuminated fluorophores are able to emit simultaneously, thereby allowing each one to be localized individually with a precision below the diffraction limit. To do so, a strong continuous excitation light source is used to instantly convert most of the fluorophores into a temporary dark state, resulting in only a few being able to switch stochastically to an active state and fluoresce^9^,^10^. The microscope records the precise position of the fluorophores over a series of imaging cycles. Because of the fact that in our setup each colour needs to be imaged sequentially, we have not been able to simultaneously observe two genomic probes of the genome marked by FISH, as the second probe is not resistant to two consecutive sessions of strong illumination. To overcome this technical limitation, we combined one genomic probe marked by FISH with the immunofluorescence of the protein P1 adhesin of the AO. By observing the genomic probe first, and then in a second session the AO by immunofluorescence, we were able to overcome the problem of photobleaching. As we found that the midpoint probe was close to the AO, we could therefore deduce the distances between Ori-midpoint, right-midpoint and left-midpoint, by estimating their median distances to the AO minus the median midpoint-AO distance.

*DNA probe preparation for FISH*. Standard PCRs were performed with genomic DNA to amplify seven regions of interest using the following pairs of primers Ori (F_Ori, R_Ori), midpoint (F_midpoint, R_midpoint), Right (F_90C, R_90C), Left (F_270C, R_270C), N1 (F_N1, R_N1), N2 (F_N2, R_N2) and N3 (F_N3, R_N3) (Supplementary Table 3). The different amplified fragments were labelled by adapting the protocol from the Random Primed DNA Labeling Kit (Roche). Briefly, the probes were denatured by incubating 10 min at 100 °C and mixed with the following reagents: 5 μl of 10 × Klenow buffer, 0.25 μl of 100 mM dATP, dCTP, dGTP, 0.16 μl of 100 mM dTTP, 2.5 μl of 1 mM ChromaTide Alexa Fluor 568-5-dUTP (Life Technologies), 1.6 μl of 3 μg μ^−**1**^ l^−**1**^ random hexamers and 0.25 μl of 10 U μ^−**1**^ l^−**1**^ Klenow fragment, before O/N incubation at 37 °C. The reaction was stopped by adding 2 μl of 0.2 M EDTA and incubated for 10 min at 65 °C. The labelled probes were then purified by ethanol precipitation.

*FISH labeling*. *M. pneumoniae* cells were grown in a 75 cm^2^ flask with Hayflick medium for 4 days under standard conditions. After 4 days, the medium was removed and cells were re-suspended in 5 ml of fresh Hayflick medium, and subsequently scrapped and collected. Aggregates were then removed by passing the cells through a syringe with a G25 needle (10 × ) and a 0.45 μm filter, and mixed with 5 ml of 6% gelatin. Then, cells were grown on borosilicate coverglass slides (Thermo Scientific) for 6 h. Cells were fixed in a final concentration of 4% formaldehyde (Pierce) for 20 min at RT followed by 40 min at 4 °C, before further fixing with cold methanol at −20 °C O/N. Subsequently, after washing twice with PBS, two washes were done with 2 × SSC/Tween-20 for 5 min, then with 2 × SSC/formamide at 37 °C for 30 min. Each genomic probe was then mixed with hybridization buffer (2 × SSC, 50% formamide and 100 μg ml^−**1**^ of salmon DNA sperm) and warmed at 95 °C for 10 min. In parallel the slides were also warmed at 95 °C for 2 min before adding the probes to the slides and incubating at 42 °C O/N.

Several washes were then done: twice with 2 × SSC/50% formamide for 30 min at 37 °C, then with 2 × SSC/25% formamide for 10 min, 3 × with 2 × SSC for 10 min and finally briefly with PBS.

When immunofluorescence localization of P1 protein was required, samples were blocked during 1 h using 2% Elisa reagent Blocking solution (Roche). Then, a primary antibody from rabbit recognizing the P1 adhesin protein of the AO (Organelle 65114, provided by Prof. Richard Herrmann) was incubated for 1 h at RT with blocking solution at a 1:1,000 dilution. After three washes with 1x PBS/0.05% Tween-20 for 15 min at RT, the secondary antibody (anti-rabbit marked with Alexa Fluor 488, IgG Invitrogen Ref A11008) was added to the slides in blocking solution for 1 h at RT at a 1:3,000 dilution. After three washes with 1 × PBS/0.05% Tween-20 for 15 min at RT, the removable slide chambers were dried and mounted on glass slides using the Prolong Gold antifade reagent (Life Technologies).

### FISH imaging acquisition and processing

The super-resolution microscope used to acquire the data was a GSD Multiline TIRF microscope (Leica, Wetzlar, Germany) using the proprietary Leica software, equipped with a 1.46 NA 100 × TIRF objective and an Andor iXon EMCCD camera. We processed the data using rapidSTORM and used PALMsiever^69^ for filtering and rendering. Finally, using Fiji, the distances were calculated between the centre of mass of the observed probes.

Additional images were taken on a Nikon N-STORM system in dSTORM mode under the corresponding excitations for the two channels (Supplementary Fig. 9). 3D information was generated using an astigmatic lens in the camera lightpath while illuminating the sample in a very shallow angle (Hilo illumination). The 3D localization data were generated using the THUNDERSTORM software package for Fiji^70^ using channel-specific 3D calibration files generated on the N-STORM system and subsequently filtered for low uncertainty and 3D density inside the software. 3D localizations were plotted into 20 nm pixels in *xy*, with a *z*-spacing of 50 nm.

### Domain detection on Hi-C contact maps

The filtered raw HpaII matrix at 3 kb resolution was used for domain detection. In addition, we computed the alignment of domain borders across the 5 HpaII replicates (Supplementary Fig. 7). First, the TADbit program^16^,^47^,^48^ normalized the matrix with a single iteration of ICE. Then TADbit returned the optimal segmentation of the chromosome under BIC-penalized likelihood. The algorithm for the domains detection uses a change-point algorithm, inspired by methods used to detect copy number variations in CGH experiments. The model assumes that counts have a Poisson distribution and that the expected value of the counts decreases as a power-law to the linear distance on the chromosome^48^. We have provided the script used to reproduce the analysis (Supplementary Software).

### Co-expression levels analysis (RNA-seq)

To analyse the level of basal coordination of transcription between all pairs (i,j) of genes, a co-expression tendency was computed for the 869 *M. pneumoniae* genes across 141 conditions (282 samples), that quantifies the tendency of the expression of the two genes to systematically vary in parallel^53^. More specifically, it determines the difference between the number of pairs of samples for which the two genes co-vary and the number for which they vary in the opposite direction, normalized by the total number of possible pairs of samples. Compared with other related correlation measures, such as the Pearson correlation or the biweight mid-correlation, this technique is particularly well suited to highlight the basal coordination of genes, irrespective of the large shifts in expression levels that may occur in some of the conditions.

We performed analyses of co-expression tendency using the co-expression matrices from both the Pearson correlation and the basal correlation. Since both matrices led to the same findings, we only presented in the manuscript the results based on the Pearson correlation. We have provided the two co-expression matrices along with the script used for the analysis (Supplementary Software).

### HpaII sites number on domain borders

We computed the number of sites on the 88 domain borders, with each domain border being defined by two bins, the last bin of the previous domain and the first bin of the next domain. We evaluated the significance using a permutation test, where all 88 domain border positions were shifted across the genome, while conserving both the size and number of domains. We obtained a number of sites per domain border for each permutation that we could compare to the original case. We obtained an empirical *P* value, calculated as the ratio between the number of values that are higher than or equal to the observed value in the original domain border case (Supplementary Methods).

### High co-expression levels within domains

We computed the mean co-expression levels between pairs of genes within the same domain, compared with pairs of genes where the first gene is localized within one domain and the second gene in a different domain. Then we computed the Mann–Whitney test *P* value to compare the two distributions (Supplementary Methods).

### Low co-expression levels across domain borders

To assess whether there was a significant low co-expression across the domain borders, we performed a permutation test, where all domain border positions were shifted across the genome while conserving both the size and number of genomic domains. Then, for each permutation, we calculated the mean co-expression levels of the genes present in the domain borders. Finally, we computed the empirical *P* value as the ratio between the number of values that are lower than or equal to the observed value in the original domain border case (Supplementary Methods).

### Data availability

The Hi-C data is already public with accession numbers: E-MTAB-3721 at https://www.ebi.ac.uk/arrayexpress/experiments/E-MTAB-3721/. The RNA-seq data is also already public with accession numbers: E-MTAB-3771, E-MTAB-3772 and E-MTAB-3773.

## Additional information

**How to cite this article:** Trussart, M. *et al*. Defined chromosome structure in the genome-reduced bacterium *Mycoplasma pneumoniae*. *Nat. Commun.* **8,** 14665 doi: 10.1038/ncomms14665 (2017).

**Publisher's note:** Springer Nature remains neutral with regard to jurisdictional claims in published maps and institutional affiliations.

## Supplementary Material

Supplementary Information Supplementary Figures, Supplementary Tables and Supplementary Methods

Supplementary Data 1 : List of DNA-binding proteins with their average copy numbers per basepair of DNA for M. pneumoniae, E. Coli and B. subtilis.

Supplementary Movie 1 3D model of the first cluster of M. pneumoniae genome models.

Supplementary Movie 2 3D reconstruction of a M. pneumoniae cell from EM imaging.

Supplementary Movie 3 3D reconstruction of a M. pneumoniae chromosome from DAPI staining and 3D-SIM imaging.

Supplementary Software We provide a folder containing all the data and scripts used to reproduce the analysis of identification of chromosomal interactions domains (CIDs). 1. Matrices_for_TADbit: contains the input raw and normalized matrices of the sum of the five HpaII replicates described in Figure 4a, of the HpaII with novobiocin described in Figure 5a, and of each of the five HpaII replicates, to calculate the CIDs borders. 2. Script_TADbit: contains the python script, iPython notebook script and html version, used in TADbit to detect the CIDs borders, and is saved in the Output_CIDs_detected/ directory. 3. Coexpression_Data: contains the two co-expression matrices (basal and Pearson) with their corresponding genes, as well as the co-expression for all genes pairs to compute the coexpression per CIDs. 4. Script_coexpression_CIDs: contains the R script used to compute the absolute mean coexpression of pairs of genes within and between CIDs.

Peer Review File

## Figures and Tables

**Table 1:** List of assigned transcription factors, sigma factors and structural proteins and essentiality with three distinct categories: essential (E), non-essential (NE) and fitness (F).

**Gene number**	**Gene name**	**Protein name**	**Essentiality**^44^
*MPN002*	*cbpA*	Curved DNA-binding protein CbpA	F
*MPN003*	*gyrB*	DNA gyrase subunit B	E
*MPN004*	*gyrA*	DNA gyrase subunit A	E
*MPN122*	*parB*	DNA topoisomerase 4 subunit B	E
*MPN123*	*parC*	DNA topoisomerase 4 subunit A	E
*MPN124*	*hrcA*	Heat-inducible transcription repressor hrcA	E
*MPN229*	*ssbA*	SSB-binding ssDNA	E
*MPN239*	*gntR*	Probable HTH-type transcriptional regulator gntR	E
*MPN241*	*whiA*	Transcription factor with WhiA C-terminal domain	F
*MPN266*	*spxA*	Transcriptional regulator Spx	E
*MPN275*	*ybaB*	DNA-binding protein, YbaB/EbfC family	F
*MPN294*	*araC*	AraC-like transcriptional regulator	NE
*MPN332*	*lon*	ATP-dependent protease La (EC 3.4.21.53)	E
*MPN352*	*sigA*	RNA polymerase sigma factor rpoD (Sigma-A) (EC 2.7.7.6)	E
*MPN424*	*ylxM*	Putative helix-turn-helix protein, YlxM/p13-like protein	NE
*MPN426*	*smc*	SMC family, chromosome/DNA binding/protecting functions	E
*MPN478*	*yrbC*	YebC family protein (transcription factor of the tetR family)	E
*MPN529*	*ihf*	Histone-like bacterial DNA-binding protein	F
*MPN554*	ssbB	Putative single-stranded DNA-binding protein	E
*MPN572*	*pepA*	Probable cytosol aminopeptidase (EC 3.4.11.1) (leucine aminopeptidase) (LAP)	E
*MPN608*	*phoU*	Transcriptional regulator involved in phosphate transport system	E
*MPN626*	*mpn626*	Alternative sigma factor	NE
*MPN686*	*dnaA*	Chromosomal replication initiator protein dnaA	E

E, essential; F, fitness; LAP, leucine aminopeptidase; NE, non-essential; ssDNA, single-stranded DNA^44^.
