# Supplementary material for: Defined chromosome structure in the genome-reduced bacterium Mycoplasma pneumoniae
Source: Nat Commun. 2017 Mar 8;8:14665. doi: 10.1038/ncomms14665 (PMC5344976; doi:10.1038/ncomms14665)
Supplement: Supplementary Information — Supplementary Figures, Supplementary Tables and Supplementary Methods [file ncomms14665-s1.pdf]

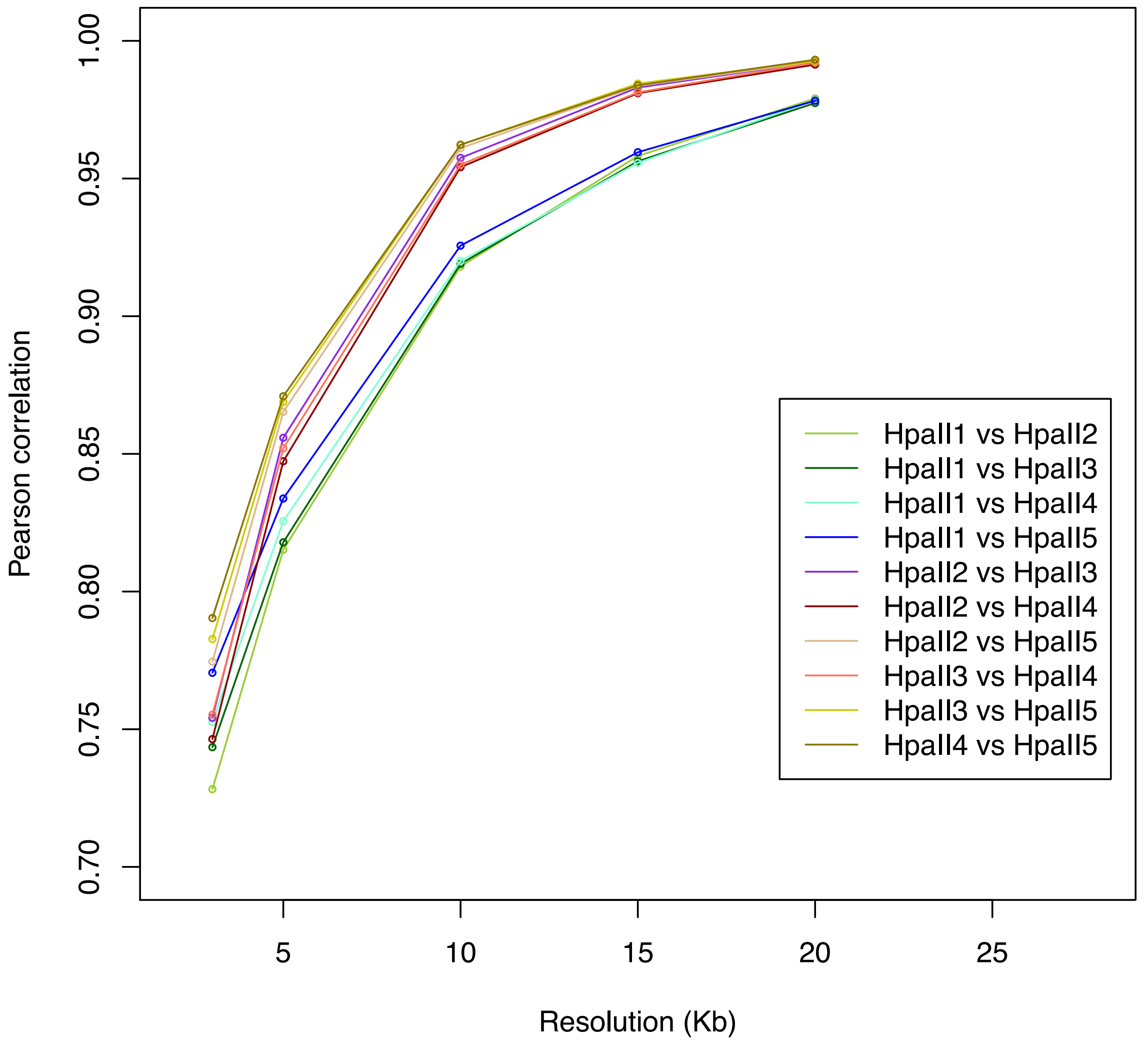

Supplementary Figure 1: Pearson correlation between HpaII replicate datasets at different resolutions.

Comparison of normalized and filtered Hi-C matrices of 5 HpaII replicate datasets across 3, 5, 10, 15 and 20 kb resolutions, shown in the x-axis, with Pearson correlation, shown in the y-axis.

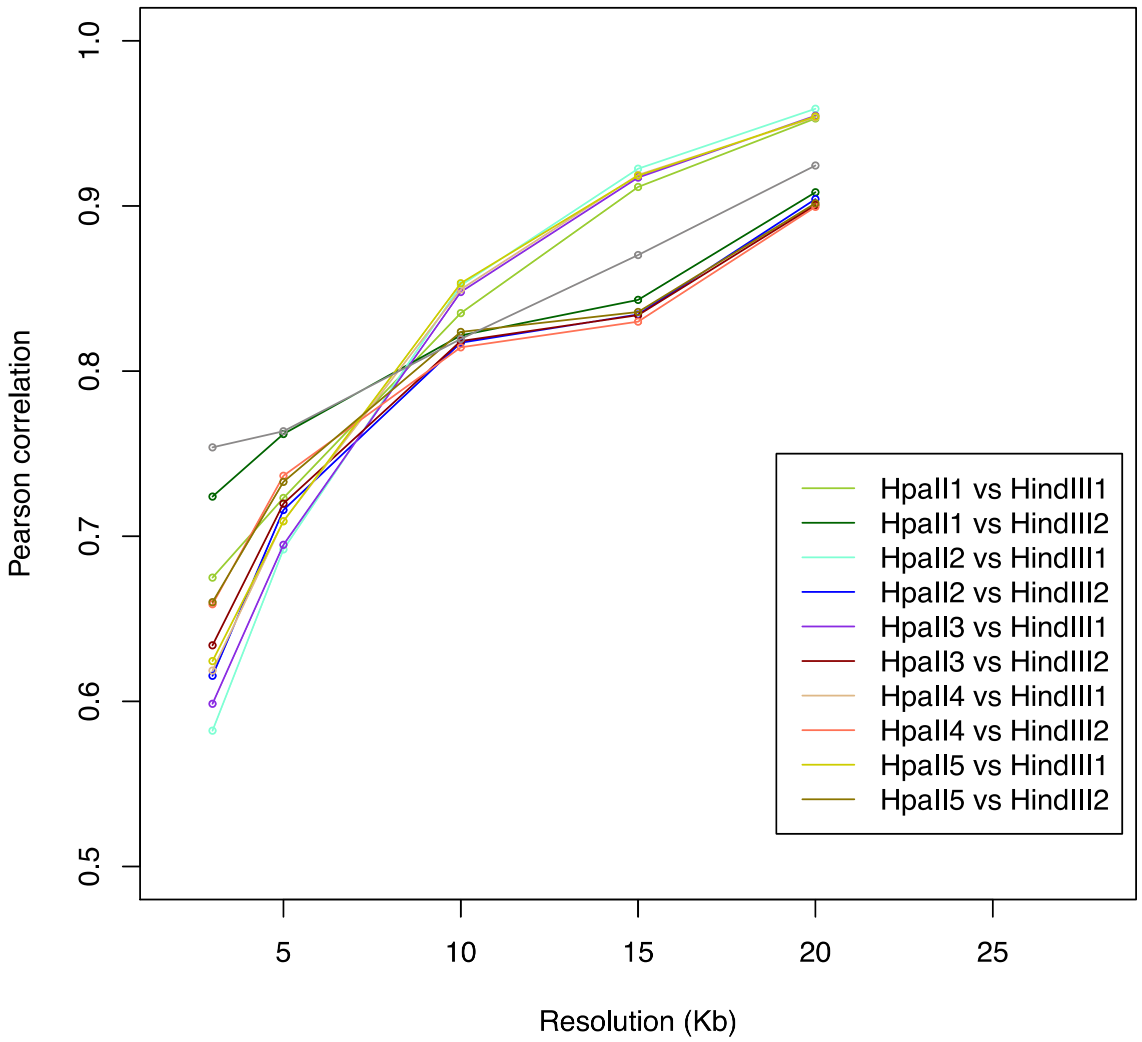

<u>Supplementary Figure 2</u>: Pearson correlation between HpaII and HindIII replicate datasets at different resolutions.

Comparison of normalized and filtered Hi-C matrices of 2 HindIII replicates and 5 HpaII replicates datasets across 3, 5, 10, 15 and 20 kb resolutions, shown in the x-axis, with Pearson correlation, shown in the y-axis.

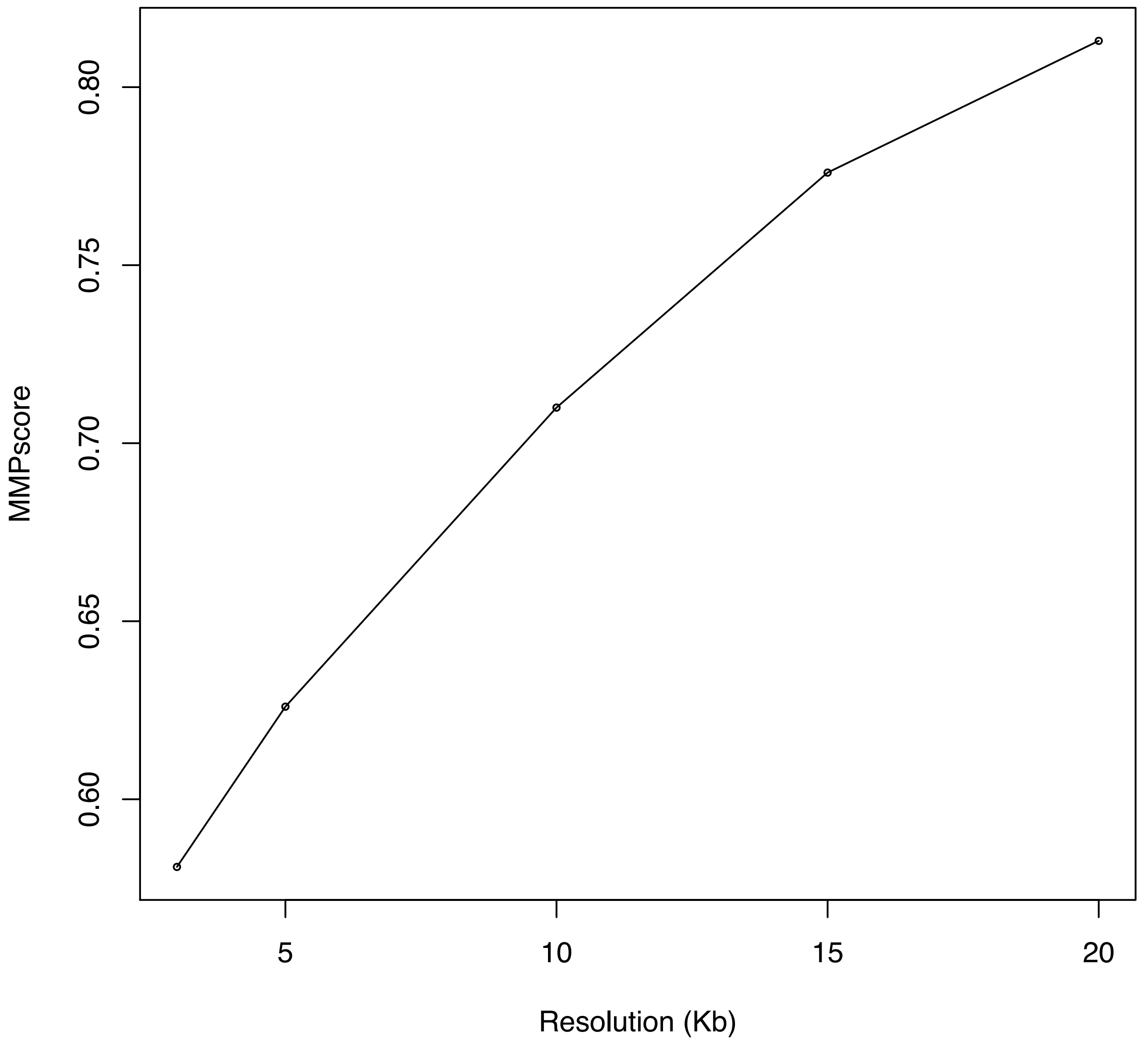

<u>Supplementary Figure 3</u>: MMP score at different resolutions for the HpaII dataset.
MMP score of the sum of the normalized and filtered 5 HpaII replicates datasets was computed, as shown in the y-axis, across 3, 5, 10, 15 and 20 kb resolutions, shown in the x-axis.

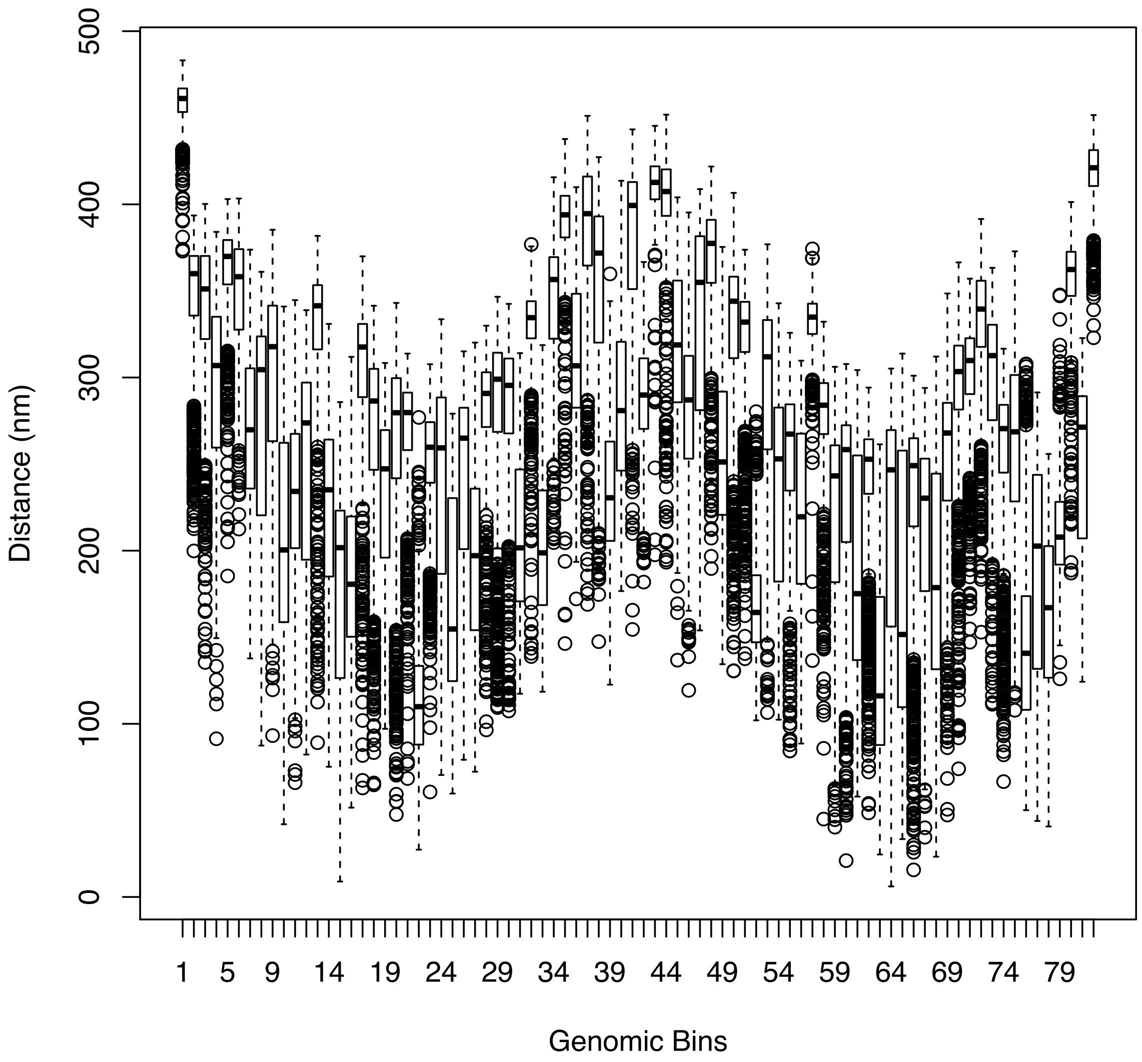

<u>Supplementary Figure 4</u>: Distance to center of mass across all models.
In all models, the center of mass was computed and the Euclidian distance of each genomic bin to the center of mass was calculated, as shown in the y-axis.

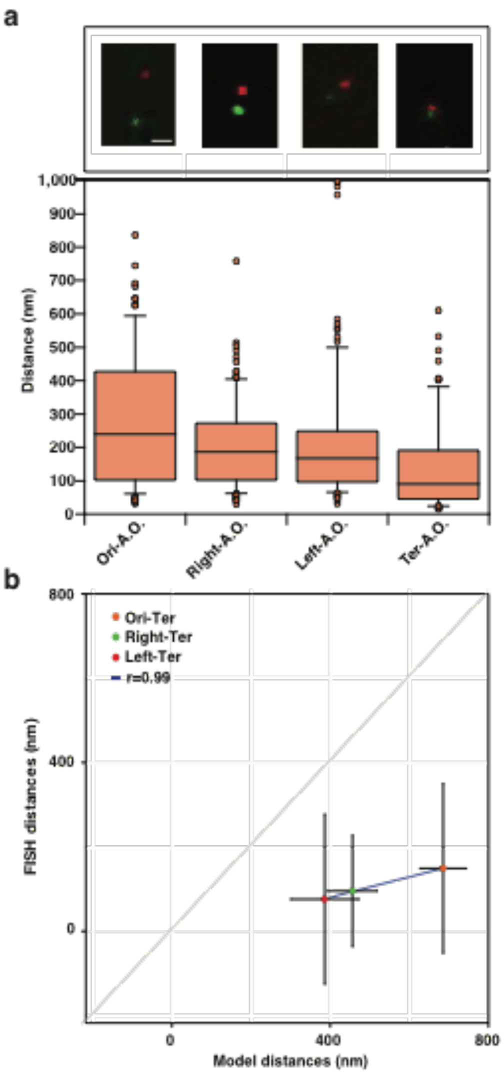

<u>Supplementary Figure 5:</u> Validation of 3D models with super-resolution imaging of cells at exponential growth phase.

(a) (Top) FISH imaging with red (Alexa Fluor 568) indicating the genomic probes Ori, Right, Midpoint, Left, N1, N2 and N3 respectively, and green (Alexa Fluor 488) representing the P1 adhesin attachment organelle protein. Bar, 200nm. (Bottom) Boxplot distribution and median distances estimated between the genomic probes and AO over 100 cells. (b) Ori-Midpoint, N1-Midpoint, Right-Midpoint, N2-Midpoint, N3-Midpoint and Left-Midpoint estimated distances from chromosome models in the x-axis and experimental FISH imaging in the y-axis. Black lines indicate the variability within the estimated distribution.

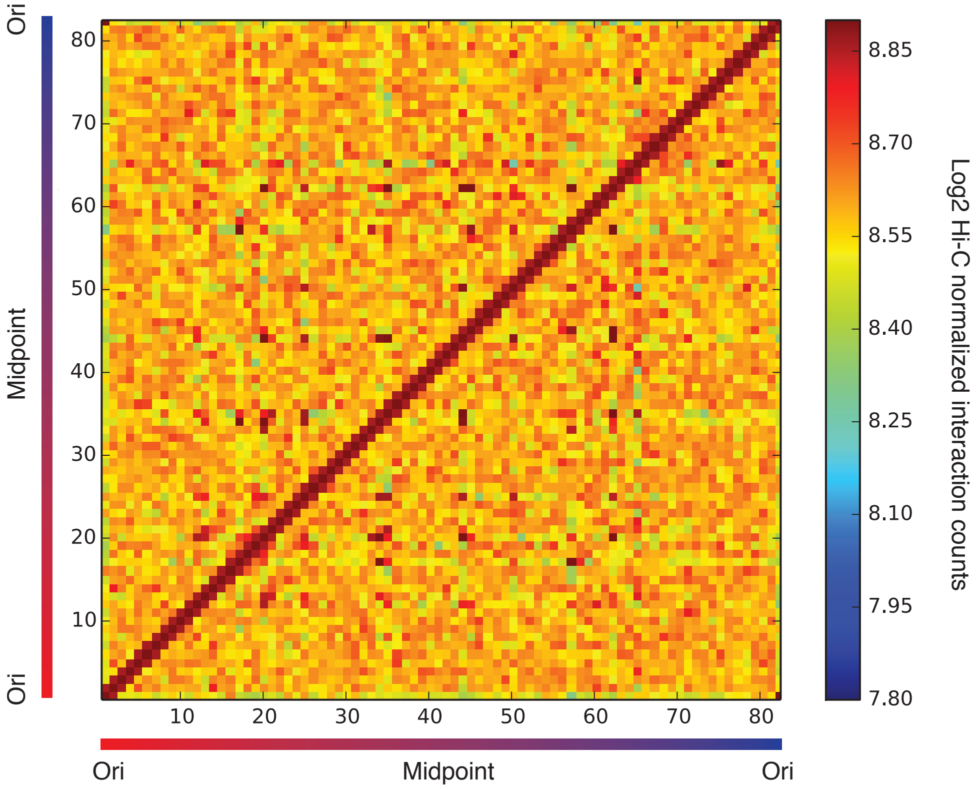

Supplementary Figure 6: Normalized HpaII Hi-C contact map of *M. pneumoniae*, in exponential phase at a 10 kb resolution. The frequency of interactions between a given pair of bins is found at the intersection of the row and column corresponding to those bins. The color of the contact map, from blue to red, indicates the log2 contact frequency. The bar underneath indicates the genome position with Ori being located at a genome coordinate of 0 and Midpoint located at ~ 400 kb.

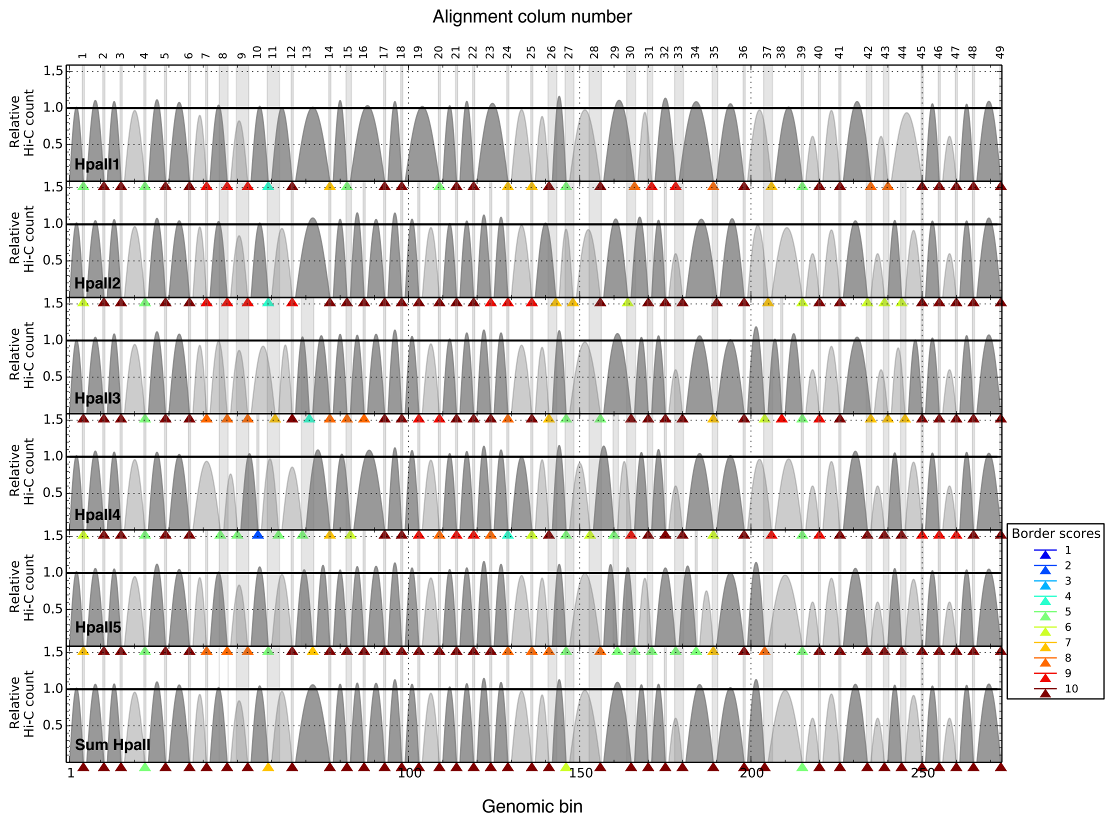

<u>Supplementary Figure 7</u>: Alignment of domain borders across 5 HpaII replicates. TADbit aligns the predicted boundaries of the 5 different replicates as well as the sum of 5 replicates and computes the alignment quality by comparing randomized boundaries of the same size, over the chromosome.

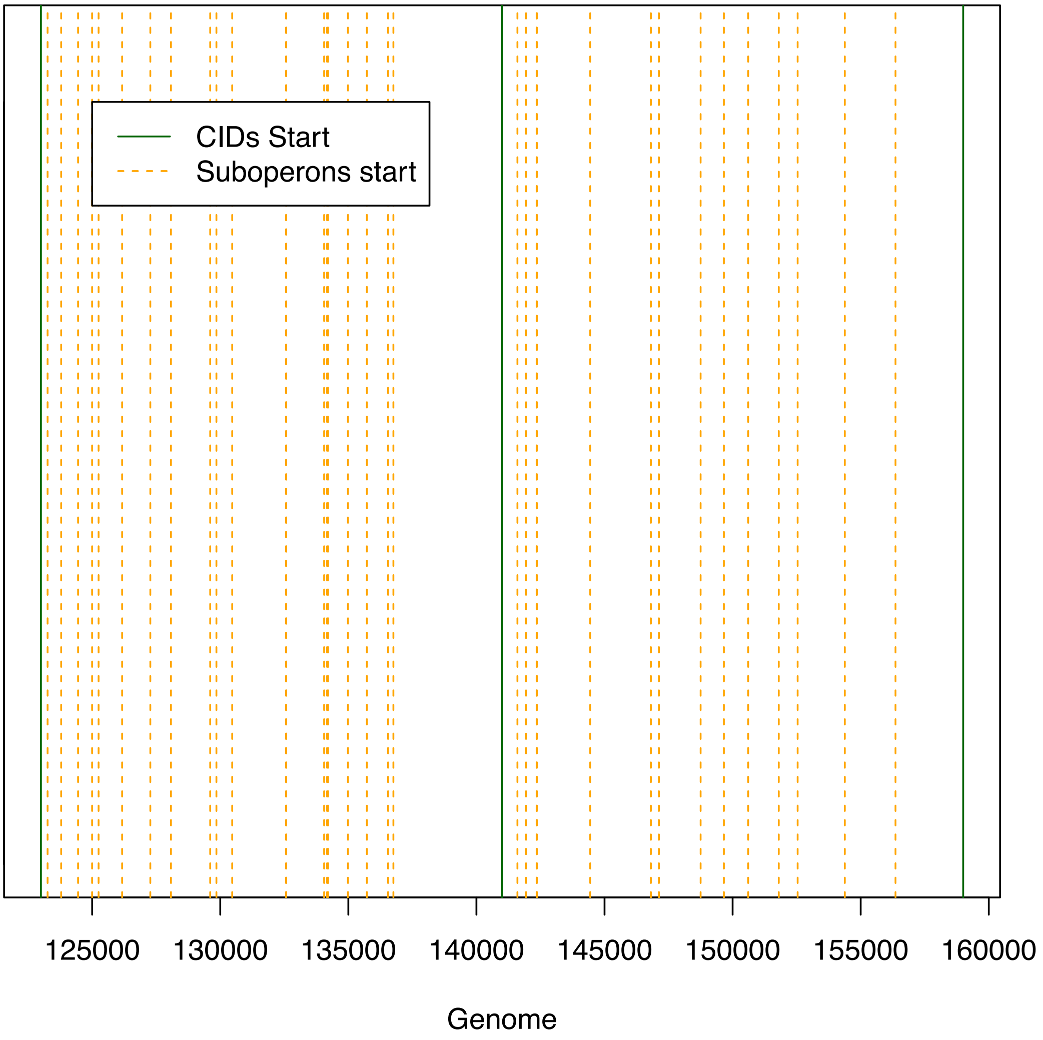

<u>Supplementary Figure 8</u>: Distribution of suboperons across CIDs 8 and 9.
A vertical green line shows the starting position of CIDs 8 and 9. In total there are 20 and 14 suboperons respectively in each domain, and a dashed orange vertical line shows their starting positions.

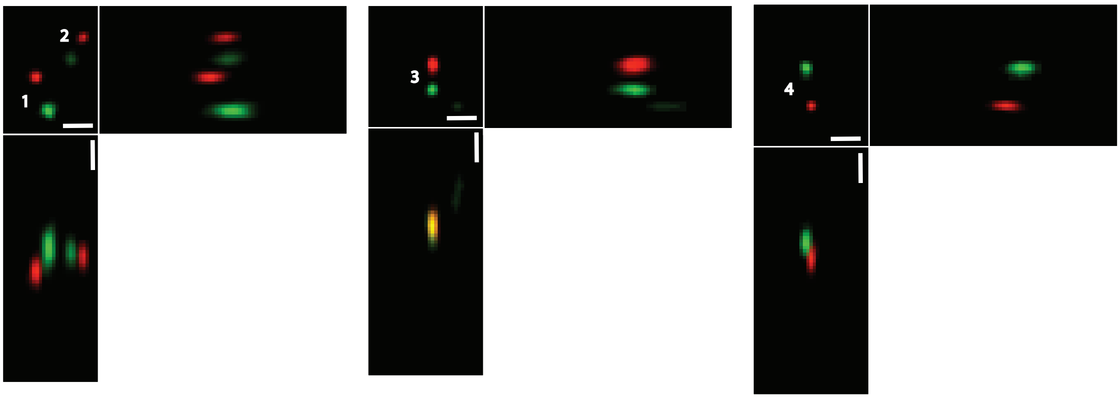

Distance (nm)	XY	XYZ
1	231	276
2	158	159
3	162	162
4	244	262

Supplementary Figure 9: Validation with another localization microscope system (Nikon N-STORM) in 3D.

3D dSTORM images of Mycoplasma cells labeled with red (Alexa Fluor 568) indicating the genomic probe Midpoint and green (Alexa Fluor 488) representing the P1 adhesin attachment organelle protein. Bar, 200 nm. The data are shown as sum projections in XY, XZ and YZ orientations. Distances between the points were calculated in the image plane (XY) and in 3D (XYZ).

Supplementary Table 1: MMP scores at 10 kb for both HindIII and HpaII datasets. MMP scores of the normalized and filtered datasets were computed using the 10 kb resolution data.

Sample	MMP score (10 kb)
HindIII1	0.738
HindIII2	0.745
HpaII1	0.741
HpaII2	0.712
HpaII3	0.728
HpaII4	0.731
HpaII5	0.729

Supplementary Table 2: Hi-C datasets statistics. Total read numbers and interaction numbers after filtering, obtained with the hiclib Python library.

Enzyme	HpaII Stat Rep.1	HpaII Stat Rep.2	HpaII Stat Rep.3	HpaII Stat Rep.4	HpaII Stat Rep.5
**Number of total reads**	111,545,820	149,906,315	150,677,819	158,093,084	154,594,333
**Total DS+SS (Valid + single sided)**	101,659,833	142,065,016	143,319,030	150,748,071	147,837,469
**TotalDSReads**	87,628,419	128,287,667	129,834,572	137,393,645	134,947,861
**SSReadsRemoved (Single sided)**	14,031,414	13,777,349	13,484,458	13,354,426	12,889,608
**SameFragmentReadsRemoved**	74,681,589	110,937,423	112,672,861	120,023,121	116,358,185
**Self-Circles**	2,192,507	3,224,919	2,843,319	1,315,820	1,103,370
**DandlingEnds**	72,449,132	107,701,113	109,815,810	118,685,242	115,226,213
**error**	39,950	11,391	13,732	22,059	28,602
**ExtraDandlingEndsRemoved**	2,870,650	4,127,721	4,861,994	4,772,150	5,868,642
**ValidPairs**	10,076,180	13,222,523	12,299,717	12,598,374	12,721,034
**StartNearRsiteRemoved**	6,225,413	5,332,067	5,052,064	4,191,014	3,955,537
**DuplicatesRemoved**	363,527	1,073,862	1,250,543	2,399,999	1,162,464
**RemovedLargeSmallFragments**	115,437	297,651	281,856	357,640	510,935
**RemovedFromExtremeFragment**	82,086	181,453	161,425	125,180	188,744
**Final interaction numbers after hiclib filtering**	3,289,717	6,337,490	5,553,829	5,524,541	6,903,354

Enzyme	HindIII Stat Rep.1	HindIII Stat Rep.2	HpaII Stat Nov.	HpaII Exp. Rep.1	HpaII Exp Rep.2	HpaII Exp Rep. 3
**Number of total reads**	107,768,945	186,752,960	72,057,609	159,272,416	144,273,685	161,313,296
**Total DS+SS (Valid + single sided)**	93,459,330	170,556,442	56,735,102	143,749,485	136,020,148	151,544,084
**TotalDSReads**	74,812,770	35,974,512	64,704,336	129,159,217	126,629,191	141,406,360
**SSReadsRemoved (Single sided)**	18,646,560	134,581,930	7,969,234	14,590,268	9,390,957	10,137,724
**SameFragmentReadsRemoved**	67,108,112	170,556,442	51,064,313	119,911,082	121,692,718	136,943,109
**Self-Circles**	11,401,814	28,739,969	519,101	726,858	1,082	900
**DandlingEnds**	55,449,411	15,780,913	50,507,874	119,165,984	121,685,229	136,936,687
**error**	256,887	12,793,947	37,338	18,240	6,407	5,522
**ExtraDandlingEndsRemoved**	1,064,607	165,109	1,525,812	3,970,743	2,141,389	2,332,656
**ValidPairs**	6,640,051	684,506	4,144,977	5,277,392	2,795,084	2,130,595
**StartNearRsiteRemoved**	345,088	495,349	2,797,304	3,696,735	1,457,646	1,092,704
**DuplicatesRemoved**	4,857,027	2,915,725	111,515	282,447	111,401	70,204
**RemovedLargeSmallFragments**	22,662	13,698	139,940	161,556	6,651	5,586
**RemovedFromExtremeFragment**	53,431	89,364	183,77	19,901	76,680	59,128
**Final interaction numbers after hiclib filtering**	1,361,843	3,035,901	1,077,841	1,116,753	1,142,706	902,973

Supplementary Table 3: Regions of interest marked by FISH. Primer sequences and respective positions of the seven regions marked by FISH.

**Primers**	**Annotation**	**Start position**	**End position**	**Genomic Sequence**
F_Ori	Ori forward strand	1	26	TATTTACCGACGAAATTAATACCATC
R_Ori	Ori reverse strand	974	1000	TTTTGTTTTGACTAAAAGAGTTTGATC
F_90C	Right forward strand	204000	204020	TTGCACCAACTCCAGCAAGAC
R_90C	Right reverse strand	204974	205000	TGCTTGTCAATCATGTACTCAATTAAC
F_Mid	Midpoint forward strand	390000	390021	CGTAACATAAAAGAAGCACGTG
R_Mid	Midpoint reverse strand	390982	391000	GTTGTTTAGCGCGGGCTTC
F_270C	Left forward strand	612000	612016	CAAGCGCTCGCCTGGTC
R_270C	Left reverse strand	612971	613000	AATTTGAACAATTTCAACTAATTTATCAAC
F_N1	N1 forward strand	99887	99906	AAGATGGACACCAAACAAAC
R_N1	N1 reverse strand	100755	100737	CCGGGCATCCAAAAGGTAA
F_N2	N2 forward strand	299892	299911	AACGGGAAACCATCCAAAAG
R_N2	N2 reverse strand	300844	300826	GTGGTGGTGTTTTTACCGA
F_N3	N3 forward strand	499920	499939	ACATTTCACTCGATCACGAC
R_N3	N3 reverse strand	500767	500750	CATACAGCATTGGATCAGT

SUPPLEMENTARY METHODS

Statistical analyses

In this study, we tested several hypotheses using p-values. If the p-value is less than (or equal to) α, then the null hypothesis is rejected in favor of the alternative hypothesis. On the other hand, if the p-value is greater than α, then the null hypothesis is not rejected. In most studies, authors refer to results being statistically significant when $p < 0.01$ or $p < 0.05$ and being statistically highly significant if $p < 0.001$.

The first statistical test we used was to compute the correlation between Hi-C replicates and is based on Pearson's product-moment correlation coefficient. It is a measure of the linear correlation between two variables. The five HpaII biological replicates have a correlation of $r > 0.91$ with a highly significant p-value< 0.0001. Similarly, the correlation between the HindIII and HpaII datasets is $r > 0.81$ with a p-value< 0.0001.

The second test we used was a permutation test to assess whether a given factor, such as the number of HpaII sites, the GC content, the number of convergent and divergent genes, or co-expression levels, is related to the domain borders. We performed the permutation test by shifting all domain border positions across the entire genome, while conserving both the size and number of genomic domains. Then, for each permutation, we calculated the mean factor

number at the domain borders. Finally, we computed the empirical p-value as the ratio between the number of values that are lower/higher than or equal to the observed value in the original domain border case. For example, we found a significant number of both convergent and divergent genes pairs with p-values= 0.026 and 0.037, respectively.

The third test we used was the two sample t-test to test whether the absolute mean co-expression of pairs of genes within and between domains are equal. A p-value of 0.0032 ($t = -3.0503$, $df = 65.658$) was obtained, indicating a true difference in means. Therefore, we were able to conclude that genes are significantly co-expressed within domains.
