# Supplementary material for: Defined chromosome structure in the genome-reduced bacterium Mycoplasma pneumoniae
Source: Nat Commun. 2017 Mar 8;8:14665. doi: 10.1038/ncomms14665 (PMC5344976; doi:10.1038/ncomms14665)
Supplement: Supplementary Software — We provide a folder containing all the data and scripts used to reproduce the analysis of identification of chromosomal interactions domains (CIDs). 1. Matrices_for_TADbit: contains the input raw and normalized matrices of the sum of the five HpaII replicates described in Figure 4a, of the HpaII with novobiocin described in Figure 5a, and of each of the five HpaII replicates, to calculate the CIDs borders. 2. Script_TADbit: contains the python script, iPython notebook script and html version, used in TADbit to detect the CIDs borders, and is saved in the Output_CIDs_detected/ directory. 3. Coexpression_Data: contains the two co-expression matrices (basal and Pearson) with their corresponding genes, as well as the co-expression for all genes pairs to compute the coexpression per CIDs. 4. Script_coexpression_CIDs: contains the R script used to compute the absolute mean coexpression of pairs of genes within and between CIDs. [file ncomms14665-s6.zip › 2017_Trussart_et_al_Coexpression_CIDs_borders/2.Script_TADbit/CIDs_detection.html]

[]


In [1]:

```
%matplotlib inline
############################################# 3 KB ###############################################################
####### Upload Hi-C data #########
from pytadbit import Chromosome
my_chrom = Chromosome(name='Mpn',centromere_search=False)
## HpaII1 replicate
my_chrom.add_experiment("hpaII1_stat",resolution=3000,hic_data='/Users/marie/Data/Hi-C_Data/Coexpression_CIDs_borders/1.Matrices_for_TADbit/HpaII1_Stat_raw_filt_3kb.txt',norm_data='/Users/marie/Data/Hi-C_Data/Coexpression_CIDs_borders/1.Matrices_for_TADbit/HpaII1_Stat_norm_filt_3kb.txt',enzyme='HpaII')
exp=my_chrom.experiments["hpaII1_stat"]
my_chrom.find_tad(['hpaII1_stat'], verbose=True,batch_mode=False)
my_chrom.experiments["hpaII1_stat"]
my_chrom.visualize("hpaII1_stat", paint_tads=True,normalized=True)
## HpaII2 new replicate
my_chrom.add_experiment("hpaII2_stat",resolution=3000,hic_data='/Users/marie/Data/Hi-C_Data/Coexpression_CIDs_borders/1.Matrices_for_TADbit/HpaII2_Stat_raw_filt_3kb.txt',norm_data='/Users/marie/Data/Hi-C_Data/Coexpression_CIDs_borders/1.Matrices_for_TADbit/HpaII2_Stat_norm_filt_3kb.txt',enzyme='HpaII')
exp1=my_chrom.experiments["hpaII2_stat"]
my_chrom.find_tad(['hpaII2_stat'], verbose=True,batch_mode=False)
my_chrom.experiments["hpaII2_stat"]
my_chrom.visualize("hpaII2_stat", paint_tads=True,normalized=True)
## HpaII3 new replicate
my_chrom.add_experiment("hpaII3_stat",resolution=3000,hic_data='/Users/marie/Data/Hi-C_Data/Coexpression_CIDs_borders/1.Matrices_for_TADbit/HpaII3_Stat_raw_filt_3kb.txt',norm_data='/Users/marie/Data/Hi-C_Data/Coexpression_CIDs_borders/1.Matrices_for_TADbit/HpaII3_Stat_norm_filt_3kb.txt',enzyme='HpaII')
exp2=my_chrom.experiments["hpaII3_stat"]
my_chrom.find_tad(['hpaII3_stat'], verbose=True,batch_mode=False)
my_chrom.experiments["hpaII3_stat"]
my_chrom.visualize("hpaII3_stat", paint_tads=True,normalized=True)
## HpaII4 new replicate
my_chrom.add_experiment("hpaII4_stat",resolution=3000,hic_data='/Users/marie/Data/Hi-C_Data/Coexpression_CIDs_borders/1.Matrices_for_TADbit/HpaII4_Stat_raw_filt_3kb.txt',norm_data='/Users/marie/Data/Hi-C_Data/Coexpression_CIDs_borders/1.Matrices_for_TADbit/HpaII4_Stat_norm_filt_3kb.txt',enzyme='HpaII')
exp3=my_chrom.experiments["hpaII4_stat"]
my_chrom.find_tad(['hpaII4_stat'], verbose=True,batch_mode=False)
my_chrom.experiments["hpaII4_stat"]
my_chrom.visualize("hpaII4_stat", paint_tads=True,normalized=True)
## HpaII5 new replicate
my_chrom.add_experiment("hpaII5_stat",resolution=3000,hic_data='/Users/marie/Data/Hi-C_Data/Coexpression_CIDs_borders/1.Matrices_for_TADbit/HpaII5_Stat_raw_filt_3kb.txt',norm_data='/Users/marie/Data/Hi-C_Data/Coexpression_CIDs_borders/1.Matrices_for_TADbit/HpaII5_Stat_norm_filt_3kb.txt',enzyme='HpaII')
exp4=my_chrom.experiments["hpaII5_stat"]
my_chrom.find_tad(['hpaII5_stat'], verbose=True,batch_mode=False)
my_chrom.experiments["hpaII5_stat"]
my_chrom.visualize("hpaII5_stat", paint_tads=True,normalized=True)
## Combined All replicates 
my_chrom.add_experiment("sum_all_rep_stat",resolution=3000,hic_data='/Users/marie/Data/Hi-C_Data/Coexpression_CIDs_borders/1.Matrices_for_TADbit/All_5HpaII_Stat_raw_filt_3kb.txt',norm_data='/Users/marie/Data/Hi-C_Data/Coexpression_CIDs_borders/1.Matrices_for_TADbit/All_5HpaII_Stat_norm_filt_3kb.txt',enzyme='HpaII')
exp5=my_chrom.experiments["sum_all_rep_stat"]
my_chrom.find_tad(['sum_all_rep_stat'], verbose=True,batch_mode=False)
my_chrom.experiments["sum_all_rep_stat"]
my_chrom.visualize("sum_all_rep_stat", paint_tads=True,normalized=True)
## Novobiocin
my_chrom.add_experiment("nov_stat",resolution=3000,hic_data='/Users/marie/Data/Hi-C_Data/Coexpression_CIDs_borders/1.Matrices_for_TADbit/HpaII_Nov_Stat_raw_filt_3kb.txt',norm_data='/Users/marie/Data/Hi-C_Data/Coexpression_CIDs_borders/1.Matrices_for_TADbit/HpaII_Nov_Stat_norm_filt_3kb.txt',enzyme='HpaII')
exp6=my_chrom.experiments["nov_stat"]
my_chrom.find_tad(['nov_stat'], verbose=True,batch_mode=False)
my_chrom.experiments["nov_stat"]
my_chrom.visualize("nov_stat", paint_tads=True,normalized=True)
```

```
/Users/marie/Softwares/anaconda/lib/python2.7/site-packages/pytadbit/parsers/hic_parser.py:113: UserWarning: WARNING: non integer values
  warn('WARNING: non integer values')
```

In [2]:

```
#### Alignment
ali=my_chrom.align_experiments(names=["hpaII1_stat","hpaII2_stat","hpaII3_stat","hpaII4_stat","hpaII5_stat","sum_all_rep_stat"])
print ali
ali.draw(normalized=True)
score, pval = my_chrom.align_experiments(randomize=True, rnd_method="interpolate",
                                         rnd_num=1000)
print 'score:', score
print 'p-value:', pval

#### Save TAD borders
exp.write_tad_borders(density=True,savedata='/Users/marie/Data/Hi-C_Data/Coexpression_CIDs_borders/2.Script_TADbit/Output_CIDs_detected/HpaII1_Stat_CID_TADbit_3kb.txt', normalized=False)
exp1.write_tad_borders(density=True,savedata='/Users/marie/Data/Hi-C_Data/Coexpression_CIDs_borders/2.Script_TADbit/Output_CIDs_detected/HpaII2_Stat_CID_TADbit_3kb.txt', normalized=False)
exp2.write_tad_borders(density=True,savedata='/Users/marie/Data/Hi-C_Data/Coexpression_CIDs_borders/2.Script_TADbit/Output_CIDs_detected/HpaII3_Stat_CID_TADbit_3kb.txt', normalized=False)
exp3.write_tad_borders(density=True,savedata='/Users/marie/Data/Hi-C_Data/Coexpression_CIDs_borders/2.Script_TADbit/Output_CIDs_detected/HpaII4_Stat_CID_TADbit_3kb.txt', normalized=False)
exp4.write_tad_borders(density=True,savedata='/Users/marie/Data/Hi-C_Data/Coexpression_CIDs_borders/2.Script_TADbit/Output_CIDs_detected/HpaII5_Stat_CID_TADbit_3kb.txt', normalized=False)
exp5.write_tad_borders(density=True,savedata='/Users/marie/Data/Hi-C_Data/Coexpression_CIDs_borders/2.Script_TADbit/Output_CIDs_detected/All_5HpaII_Stat_CID_TADbit_3kb.txt', normalized=False)
exp6.write_tad_borders(density=True,savedata='/Users/marie/Data/Hi-C_Data/Coexpression_CIDs_borders/2.Script_TADbit/Output_CIDs_detected/HpaII_Nov_Stat_CID_TADbit_3kb.txt', normalized=False)
```

```
Alignment shown in 3 Kb (6 experiments) (scores: 0 1 2 3 4 5 6 7 8 9 10)
     hpaII1_stat:|     5|    11|    16|    23|    29|    36|    41|    47|    53| ---- |    59|    66| ---- |    77|    82| ---- |    93|    98| ---- |   109|   114|   119| ---- |   129|   136|   141|   146|   156| ---- |   166|   171| ---- |   178| ---- |   189|   198|   206| ---- |   215|   220|   226|   235|   240| ---- |   250|   255|   260|   265|   273
     hpaII2_stat:|     5|    11|    16|    23|    29|    36|    41|    47|    53| ---- |    59|    66| ---- |    77|    82|    87|    93|    98|   103|   109|   114|   119|   124|   129|   136|   143|   148|   156| ---- |   164|   170|   175|   180| ---- |   190|   198|   205| ---- |   215|   220|   226|   234|   239|   244|   250|   255|   260|   265|   273
     hpaII3_stat:|     5|    11|    16|    23|    29|    36|    41|    47|    53| ---- |    61|    66|    71|    77|    82|    87|    93|    98|   103|   109|   114|   119|   124|   129|   136|   141|   146|   156| ---- |   165|   170|   175|   180| ---- |   189|   198|   204|   209|   215|   220|   226|   235|   240|   245|   250|   255|   260|   265|   273
     hpaII4_stat:|     5|    11|    16|    23|    29|    36| ---- |    45|    50|    56|    62| ---- |    69|    77|    83| ---- |    93|    98|   103|   109|   114|   119|   124|   129|   136|   141|   146|   153|   160|   165|   170|   175|   180| ---- |   189|   198|   206| ---- |   215|   220|   226|   234|   239|   244|   250|   255|   260|   265|   273
     hpaII5_stat:|     5|    11|    16|    23|    29|    36|    41|    47|    53| ---- |    59|    66|    72|    77|    82|    87|    93|    98|   103|   109|   114|   119|   124|   129|   136|   141|   146|   156|   161|   166|   171| ---- |   178|   184|   189|   198|   204| ---- |   215|   220|   226|   234|   239|   244|   250|   255|   260|   265|   273
sum_all_rep_stat:|     5|    11|    16|    23|    29|    36|    41|    47|    53| ---- |    59|    66| ---- |    77|    82|    87|    93|    98|   103|   109|   114|   119|   124|   129|   136|   141|   146|   156| ---- |   165|   170|   175|   180| ---- |   189|   198|   204| ---- |   215|   220|   226|   234|   239|   244|   250|   255|   260|   265|   273
```

```
score: Alignment shown in 3 Kb (7 experiments) (scores: 0 1 2 3 4 5 6 7 8 9 10)
     hpaII1_stat:|     5|    11|    16|    23|    29|    36|    41|    47| ---- |    53|    59|    66| ---- |    77|    82| ---- |    93|    98| ---- |   109|   114|   119| ---- |   129| ---- |   136|   141|   146|   156| ---- |   166|   171|   178| ---- |   189|   198|   206| ---- |   215|   220|   226|   235|   240| ---- |   250|   255|   260|   265|   273
     hpaII2_stat:|     5|    11|    16|    23|    29|    36|    41|    47| ---- |    53|    59|    66| ---- |    77|    82|    87|    93|    98|   103|   109|   114|   119|   124|   129| ---- |   136|   143|   148|   156| ---- |   164|   170|   175|   180|   190|   198|   205| ---- |   215|   220|   226|   234|   239|   244|   250|   255|   260|   265|   273
     hpaII3_stat:|     5|    11|    16|    23|    29|    36|    41|    47| ---- |    53|    61|    66|    71|    77|    82|    87|    93|    98|   103|   109|   114|   119|   124|   129| ---- |   136|   141|   146|   156| ---- |   165|   170|   175|   180|   189|   198|   204|   209|   215|   220|   226|   235|   240|   245|   250|   255|   260|   265|   273
     hpaII4_stat:|     5|    11|    16|    23|    29|    36| ---- |    45|    50|    56|    62|    69| ---- |    77|    83| ---- |    93|    98|   103|   109|   114|   119|   124|   129| ---- |   136|   141|   146|   153|   160|   165|   170|   175|   180|   189|   198|   206| ---- |   215|   220|   226|   234|   239|   244|   250|   255|   260|   265|   273
     hpaII5_stat:|     5|    11|    16|    23|    29|    36|    41|    47| ---- |    53|    59|    66|    72|    77|    82|    87|    93|    98|   103|   109|   114|   119|   124|   129| ---- |   136|   141|   146|   156|   161|   166|   171|   178|   184|   189|   198|   204| ---- |   215|   220|   226|   234|   239|   244|   250|   255|   260|   265|   273
sum_all_rep_stat:|     5|    11|    16|    23|    29|    36|    41|    47| ---- |    53|    59|    66| ---- |    77|    82|    87|    93|    98|   103|   109|   114|   119|   124|   129| ---- |   136|   141|   146|   156| ---- |   165|   170|   175|   180|   189|   198|   204| ---- |   215|   220|   226|   234|   239|   244|   250|   255|   260|   265|   273
        nov_stat:|     3|    11|    16|    23|    29|    37| ---- |    45|    50|    55|    62|    69| ---- |    77|    83| ---- |    93|    98| ---- |   107|   114|   119| ---- |   128|   133|   138| ---- |   148|   153|   159|   165|   171|   176|   183|   189|   197|   207| ---- |   215|   220|   226|   234|   239|   244|   250|   255|   260|   265|   273

p-value: (5.257488945578231, 0.0)
```

In []:

```

```

In []:

```

```
