# Supplementary material for: Defined chromosome structure in the genome-reduced bacterium Mycoplasma pneumoniae
Source: Nat Commun. 2017 Mar 8;8:14665. doi: 10.1038/ncomms14665 (PMC5344976; doi:10.1038/ncomms14665)
Supplement: Peer Review File [file ncomms14665-s7.pdf]

Reviewers' comments:

Reviewer #1 (Remarks to the Author):

A Summary of the key results The original article, "Defined chromosome structure in a minimal cell" from the group led by Maria Lluch-Senar and Luis Serrano is another major research project in that team's efforts to understand the systems biology of the bacterium Mycoplasma pneumoniae. This paper uses Hi-C whole genome chromosome conformation capture and high-resolution microscopy to analyze M. pneumoniae chromosome structure. Based on the long distance interactions between chromosome regions on either side of the origin of replication, computer algorithms designed to TADbit Hi-C data suggest the chromosome is structured to facilitate the M. pneumoniae chromosome segregation process. This observation and chromosome model was corroborated by EM and high resolution light microscopy studies. They report that as in other bacteria analyzed using Hi-C, the chromosome is organized into chromosome interacting domains (CIDs). Furthermore, they report that genes within those CIDs are more likely to be co-expressed than gene pairs spanning two CIDs.

B Originality and interest: The methods employed in this paper are not original; however no one has conducted this sort of study on a mycoplasma or any other bacterium with a small genome. The authors correctly cite other Hi-C studies of the bacteria Caulobacter cresentus, Escherichia coli, and Bacillus subtilis that generated models for chromosome structure and in some cases observations of CIDs. This begs the question why should yet another analysis of a bacterial chromosome structure be reported in a high profile journal like Nature Communications? The Serrano group uses M. pneumoniae, which has a small 692 gene genome in which about half of the genes are essential, as a surrogate for a minimal cell. Since 2009, his team has published a dazzling set of landmark systems biology papers on M. pneumoniae. While, they only have a very limited ability to genetically manipulate this bacterium, they have still brilliantly characterized the organism at many different levels using all the tools of modern molecular microbiology available to work with this organism. As an avid fan of their work, I was happy to get an early look at their findings. I am sure that many other biologists seeking to understand the first principles of cellular life will feel the same.

C Data & methodology: validity of approach, quality of data, quality of presentation

- Hi-C data has been used to investigate the structure of bacterial chromosomes for several species. As such, the approaches taken by these authors seem valid. Nonetheless, based on my interpretation of the data in this and other Hi-C studies of bacterial genomes, it does appear that the dynamic range of signals shown on the interaction map (Figure 1a) are much lower for M. pneumoniae than found in other studies. In Figure 1a, the colors shown on the contact map, from blue to red, indicate the log2 contact frequency for each M. pneumoniae interaction bin. The colors correspond to a range from 212 to 212.9. If I am interpreting this correctly, that means a range of less than two fold for the signal differences. The numbers shown for Hi-C interaction maps of C. crescentus, B. subtilis, and E. coli vary from 50 to over 1000 fold. Can the authors explain why the M. pneumoniae interaction data appears to be so much less dynamic? As noted in the text, the interaction patterns in figure 1a are similar to those of B. subtilis and C. crescentus but the range is much smaller.
- There should be data in supplementary information from the analyses of local interactions that partitioned the genome into 44 CIDs and then identified patterns of gene co-expression for genes inside a CID relative to genes in different CIDs.
- The sequence data should be posted in a data repository.
- Lines 745-9. Co-expression tendency based on RNA-seq expression over 282 conditions. The meaning of this statement as well as the whole topic of co-expression is unclear. The experiments that report
- Furthermore, the in press Junier paper that was listed should have been provided if it was necessary to reference it.
-

D Appropriate use of statistics and treatment of uncertainties. I am not qualified to judge this. I can say that the authors largely used some of the same statistical tests employed by other researchers that have analyzed Hi-C data from bacteria. However, it is by no means clear how many of the statistics they report were calculated or what the statistical tests are actually measuring.

E Conclusions: robustness, validity, reliability For the most part the conclusions are consistent with the results. I did not find the argument that the dearth of chromosome associated proteins encoded by M. pneumoniae meant that the folding of the DNA was not mainly driven by proteins.

F Suggested improvements: experiments, data for possible revision

• Ideally the authors would insert a large segment of stuffer DNA in either the left of right half of the genome to determine whether the terminus of genome replication is at specific sequence or rather, just the spot on the chromosome that is approximately halfway from the origin. This could be done using a transposon with a large gene free stuffer, perhaps 50 or 100 kb, or direct recombination of DNA into M. pneumoniae has been reported (Krishnakumar, et al. 2014 PMID: 24914053).
• The authors note that M. pneumoniae has few genes whose products might be expected to take part in establishing chromosome structure. On line 405 they write: As M. pneumoniae however has a limited number of copies of the histone-like IHF protein, thereby making it difficult to maintain the CID boundaries, it is likely that additional factors contribute to the formation of such domain loops. Furthermore, since M. pneumoniae only has a handful of DNA-binding proteins and very few TFs (Table 1), it is intriguing that it is capable of establishing a well-defined chromosome structure as well as maintaining CID boundaries. The list of M. pneumoniae transcription factors, sigma factors, and structural proteins (Table 1) should also include how many copies of each protein are present on average. These authors have determined this in other work. If they are adamant about this theory they could even report how many copies of each protein exist per basepair of DNA and how many exist in the organism they were comparing M. pneumoniae with, C. crescentus.

G References: appropriate credit to previous work?
Yes

Clarity and context: lucidity of abstract/summary, appropriateness of abstract, introduction and conclusions

This was a difficult paper to read and understand. In its current form, it should not be published. I do not think of Nature Communications as a specialty journal. Accordingly, a reader needs to be able to understand the data that are presented without needing to invest in reading still another paper in order to appreciate and evaluate the new paper. Furthermore, the reason that a paper on an otherwise esoteric organism that is a minor human pathogen is under consideration for Nature Communication publication is because M. pneumoniae is a model for a minimal bacterial cell. The paper needs to be written as more of a story than it is now. The only way I was able to get through it was to read very carefully the Hi-C of Caulobacter cresentus paper by Umbarger, M.A. et al. The three-dimensional architecture of a bacterial genome and its alteration by genetic perturbation. Mol. Cell 44, 252-64 (2011).

The authors use, but do not define, the term co-expression. In discussion of the phrase with my colleagues, some thought that genes in a single transcriptional unit are co-expressed. Others thought that co-expression pairs of genes transcribed at similar levels. The term needs to be described clearly. Furthermore the data used to calculate co-expression levels needs to be included in supplementary materials.

Figure 1. The last part of the Figure 1 legend states: "The color indicates the strand position, with pink being the - strand and green the + strand. (c) 3D density map representation of the first cluster of M. pneumoniae genome models with Ori and Ter represented by red and purple circles, respectively. A color tube shows the centroid model, following the same color code as the bar in (a), and the lighter color represents the space occupied by all the models in the cluster, i.e. the variability across the cluster." There is a lighter shading of the figure below the origin position. Does that indicate the model predicts the absence of chromosomal DNA in that region of the cell. If so why, I thought the terminal organelle was nearer the terminus. Also, the sentence that begins with "A color tube shows the centroid model..." I presume this is jargon to explain that the line represents the highest probability model for chromosome location. In any case, the sentence is not clear in its meaning.

Figure 4 is confusing. The legend mentions that the data in 4a is filtered and normalized. In fact at six sites the paper the data is reported as being filtered and normalized. The authors reference the Imakaev et al. paper "Iterative Correction of Hi-C Data Reveals Hallmarks of Chromosome Organization" however the process is presented as a black box. There should be an explanation of why this is done and how it changes the outcome of the analysis. The 4a diagram has domains represented as grey-filled arcs delimited by a colored line. There is no explanation why 15 of the arcs are light grey and 29 are dark grey. Because the authors felt it was necessary to show the Hi-C matrix 3kb res data (the blue section of the figure), there must be data that needs to be gleaned from the diagram, but I cannot discern what that might be. In 4c, the authors state "Detailed absolute mean co-expression distribution across the 44 domains. Point sizes are proportional to border strength. The color depicts, as before, the two cases of gene pairs within the same domain, shown in green, and gene pairs between different domains, shown in blue. " From this data, I am led to believe that at least on one side of each domain, there is a transcriptional unit with at least one gene in each domain. Otherwise, some of the domains would be represented by only green dots. Am I misinterpreting this? Finally, in part 4a Hi-C matrix interact. Freq. can be as high as 1.5 apparently. While in 4b, c, and d it is only possible to achieve a level of 1.0. These y-axis values have unclear units of measure. Interpretation is not easily possible.

Figure 5 has many of the issues listed above for Figure 4. One specific question is what is CID density, what are the units, and how is it calculated.

Figure 6 shows four different models for bacterial chromosome organization. The authors state the M. pneumoniae model was generated by TADbit software. I think this was the same software used at least for the C. crescentus modeling. Ideally, the models should be drawn so as to be more comparable. It is unclear how much of what is shown is an artistic interpretation of chromosome architecture and how much is a visual output from the TADbit software.

Reviewer #2 (Remarks to the Author):

Bacterial chromosomes are now known to be highly organized and potentially serve biological roles. Chromosome organization were widely studied in bacterial model organisms such as E. coli, B. subtilis and C. crescentus. However, the organisation in other lesser-known bacterial species have not been explored. In this manuscript, Trussart et al employed latest technologies such as chromosome conformation capture (Hi-C) and high-resolution FISH to explore the chromosome organization of a genome-reduced Mycoplasma pneumoniae. Luis Serrano and Marc Martin-Renom are experts at systems biology of M. pneumonia and at computational analysis of Hi-C data, respectively. I was eager to read this manuscript but in the end I found this manuscript immature for Nature Communications. There are not much experimental data but the authors made very bold statements.
Below are my specific points:

1. Abstract page 2 line 32-33: the authors wrote "DNA-binding proteins are central regulators of chromosome organization, however in genome-reduced bacteria their diversity is largely diminished" and they keep using this to argue that "nucleoid-associated proteins are not essential for chromosome organization Abstract page 2 line 42-44". Looking at the Table 1, clearly this is not the case. M. pneumoniae has SMC, Ihf, CbpA together with DNA topoisomerases. This is about the same number of NAPs that B. subtilis and C. crescentus has. Indeed, this is nowhere near the number of NAPs in E. coli but it could well be that new and important classes of NAPs have not been discovered yet. There are more in E. coli just because it is a very well-studied bacterium.

2. M. pneumoniae indeed has a reduced genome but they have as many NAPs (see point 1 above). That can only argue that NAPs, SMC and topoisomerases are very important (potentially for chromosome organization), so important that even genome-reduced organisms must keep them. Therefore, I don't agree with the sentence "nucleoid-associated proteins are not essential for chromosome organization Abstract page 2 line 42-44". Furthermore, in C. crescentus and B. subtillis, when smc is deleted, the less prominent secondary arm is gone suggesting that SMC is needed for a global chromosome organization. Deletion of hup again sees the reduction in short-range interactions. Perhaps, the authors should delete or deplete smc/ihf/cbpA in M. pneumoniae before concluding that they are not important for the chromosome organization in this species.

3. The authors seem to define the chromosome organization as "having domains and domain boundaries". This is just one feature of the chromosome structure and it is still too early to be certain that domains are of biological significance.

4. Result Page 7 line 134 "Although the Hi-C interaction maps obtained at exponential and stationary phase display similar features, the analysis of the chromosome structure at exponential phase could be hampered by heterogeneity, as it is not possible to synchronize M. pneumoniae. Therefore, we concentrated on the stationary phase samples". I do not agree that the Hi-C maps for exponential and stationary phase are similar at all. Looking at Supp figure 5, I don't see the less prominent secondary diagonal. It looks more like a failed Hi-C since there is nothing coming out from the background noise except for the primary diagonal which is very strong in any case anyway. The correlation between exponential phase and stationary phase samples is 0.6 (not that strong) and potentially most of the 0.6 is from the strong dominant diagonal. Could the authors revise the way they calculate the Pearson's correlation value please? Specifically, I would like them to block away the main diagonal and just compare the rest of the Hi-C maps between the two conditions. Also, please report how many reads are discarded in each step of the Hi-C analysis so readers can judge if this is indeed a failed ligation step, leading to a failed Hi-C. Right now, the authors only report the start and the final number of reads.

5. I have just looked at the Le et al 2013 Caulobacter Hi-C paper again and it seems that even in a mixed population of Caulobacter crescentus (un-synchronized), all the features of the maps (main and secondary diagonals) are still seen so I don't think that the not-so-good exponential phase map of M. pneumoniae is due to too much heterogeneity.

6. I am especially worried about using stationary phase to do Hi-C and to generalize about chromosome organisation. It is known that in E. coli, stationary cells have especially compacted chromosome/nucleoid, very different from exponential phase. Furthermore, the authors use exponential phase cells (instead of stationary phase cells) for FISH microscopy so it is just not possible to merge the two datasets to say a concrete and consistent thing about the chromosome organization in M. pneunmoniae.

7. Measuring the nucleoid volume of DAPI stained cells is a weak way to validate the model. The model does not give us much more information than the FISH data. Just by looking at the FISH data, one can already learn that ORI and TER are at the 2 opposite poles of the cells and LEFT and RIGHT are in the middle. Interestingly, the 2 arms of the chromosome in M. pneumoniae are not

twisted like in the case of C. crescentus. I believe the same group did the modelling for C. crescentus as well. This is an interesting point that could be explained by combining modelling with more FISH data of more DNA loci. Perhaps this will strengthen the manuscript?

8. Figure 4. I can hardly see the domains and domain boundaries at all. The colour choice makes it really hard to see. Perhaps the authors should present it better?

9. I don't agree that genes within each domain are co-regulated. Domain size are 15-30kb in this study, much longer than an average operon in bacteria. That makes the co-expression analysis within versus between domain faulty to start with. In this case, the co-regulation of genes is dominated by operon structure. It has little to do with chromosomal domains.

Reviewer #3 (Remarks to the Author):

The MS studies 3D genome structure in a small bacterium. This is a highly interesting scientific problem: Ample evidence has shown that not only mammalian cell nuclei but also other vertebrates and non-vertebrates maintain a highly sophisticated 3D nanostructure of their genome which is supposed to play an important role in transcription, splicing, replication and repair. From the evolutionary point of view one might speculate that such structures might have started to be formed already at the beginning of life, due to the selection pressure correlated with functional spatial organization.

The authors address this problem by combining Hi-C and super-resolution microscopy, to determine the structure of the M. pneumoniae chromosome at a 10 kb resolution. From modelling Hi-C data, they found evidence for a defined structure, with a global symmetry between the two arms connecting the opposite poles bearing the chromosomal Ori and Ter. A more refined Hi-C analysis of local structures at a 3 kb resolution indicated that the chromosome was further organized into small subdomains, with a size from 15 to 33 kb. In eukaryotes, this would correspond to clusters with a number of nucleosomes in the range around 100 - 200. Since recent findings from localization based super-resolution methods suggested that small nucleosome clusters might exist also in eukaryotes, such a finding might have a great potential interest for a better understanding of the evolution of spatial genome structure. In addition, the authors found that genes within the same domain tended to be co-regulated, suggesting that even in minimal bacteria local chromosome organization influences transcriptional regulation. This influence has been well established in mammalian cells; its existence even in minimal bacteria supports the notion of a long evolutionary history. Altogether, the findings indicate that a defined chromosomal structure is a universal feature existing throughout evolution; even nucleoid-associated proteins appear to be not essential for this.

Specific remarks:
1) Hi-C modelling: Although for the general approach a number of quotations are given, it is not clear how these principles are applied in detail to the present data set. It is recommended to provide more detailed evidence for this in the supporting material; since in principle the Hi-C data provide interaction probabilities only, not directly spatial coordinates, the modelling and its implications are of great importance.

2) The ms contains a fair amount of quite detailed conclusions read from the Hi-Ci map. While the description and basic evaluation of the Hi-C data appears to be plausible, these detailed conclusions are difficult to follow by a reader who is not a professional expert in the interpretation of such Hi-C graphs. It is suggested to give a broader justification for each of these detailed conclusions. This can be done in the supplementary material.

3) Superresolution imaging: An obvious way to reduce the ambiguity inherent in the derivation of spatial structures from Hi-C data is superresolution imaging. These novel techniques (the development of some of these approaches was honored by the 2014 Nobel prize in Chemistry) allow to extend the range of light microscopic imaging to the single molecule resolution level. Publications e.g. by the Xiaowei Zhuang lab (see quotation 9) show how effectively in particular methods of localization microscopy can be used for the study of bacteria. Towards this goal, the authors so far made little efforts, compared with the broad claim given in the abstract: They did some experiments using a commercial localization microscope (GSDIM). The quotations (58,59) they give for the method cover some aspects of localization microscopy; however, they do not describe the method actually used: while the Rust et al. paper describes the use of pairs of molecules, the Betzig paper is based on photoactivation of specific GFPs; they also do not describe the 'blinking' based localization microscopy using FISH which was successfully introduced already a number of years ago. Concerning the problem of two-color localization microscopy, three-color based localization microscopy (using human cancer cells and photostable dyes) has already been shown to work successfully in the beginning of the 2000s by a German-Israeli collaboration, achieving an intranuclear optical resolution of few tens of nm. Concerning the localization microscopy (GSDIM) based FISH results, the authors appear to evaluate the distances only; by doing so, they did not exploit the possibility to obtain not only the distances in a range much below the conventional optical resolution of ca. 200 nm but also the size of the labeled Ori/Ter regions at the nanoscale. It is recommended to provide additionally a more thorough evaluation of their GSDIM data according to the state of the art, and to compare the results obtained with the spatial predictions of the Hi-C data. At the present state, the claim made in the abstract of "combining Hi-C and super-resolution microscopy" looks somewhat too strong. To justify such a claim, in a revised ms it should be very interesting to provide localization microscopy data of a few other gene regions; e.g., it should be possible with this method to experimentally verify the finding of the Hi-Ci data of the existence of 15 - 33 kb small clusters; what is the spatial extension of such clusters? Various approaches how to achieve this by localization microscopy have been published and might be useful.

4) Discussion: For the general reader, the ms should be become much more interesting if imbedded into the general discussion of functional genome architecture and its evolution. For example, the ms makes believe that the partitioning of the mammalian genome into topologically distinct units was discovered only recently using sequencing data. While these results are highly important and relevant, the general discovery of such distinct domains has been established by light microscopy already in the 1990s. It should be helpful to add a few remarks/quotations on this. Especially interesting should be to compare the results also with functional genome nanostructure in mammalian cells as the presently most studied system.

Comments from the reviewers are shown in **bold**

Responses to the comments are shown in plain text

Reviewers' comments:

Reviewer #1 (Remarks to the Author):

A. Summary of the key results The original article, "Defined chromosome structure in a minimal cell" from the group led by Maria Lluch-Senar and Luis Serrano is another major research project in that team's efforts to understand the systems biology of the bacterium Mycoplasma pneumoniae. This paper uses Hi-C whole genome chromosome conformation capture and high-resolution microscopy to analyze M. pneumoniae chromosome structure. Based on the long distance interactions between chromosome regions on either side of the origin of replication, computer algorithms designed to TADbit Hi-C data suggest the chromosome is structured to facilitate the M. pneumoniae chromosome segregation process. This observation and chromosome model was corroborated by EM and high resolution light microscopy studies. They report that as in other bacteria analyzed using Hi-C, the chromosome is organized into chromosome interacting domains (CIDs). Furthermore, they report that genes within those CIDs are more likely to be co-expressed than gene pairs spanning two CIDs.

B. Originality and interest: The methods employed in this paper are not original; however no one has conducted this sort of study on a mycoplasma or any other bacterium with a small genome. The authors correctly cite other Hi-C studies of the bacteria Caulobacter cresentus, Escherichia coli, and Bacillus subtilis that generated models for chromosome structure and in some cases observations of CIDs. This begs the question why should yet another analysis of a bacterial chromosome structure be reported in a high profile journal like Nature Communications? The Serrano group uses M. pneumoniae, which has a small 692 gene genome in which about half of the genes are essential, as a surrogate for a minimal cell. Since 2009, his team has published a dazzling set of landmark systems biology papers on M. pneumoniae. While, they only have a very limited ability to genetically manipulate this bacterium, they have still brilliantly characterized the organism at many different levels using all the tools of modern molecular microbiology available to work with this organism. As an avid fan of their work, I was happy to get an early look at their findings. I am sure that many

other biologists seeking to understand the first principles of cellular life will feel the same.

We appreciate that this reviewer acknowledges the relevance of our work - correlating different approaches to assess whether a minimal cell with a reduced genome adopts a defined chromosome structure.

C. Data & methodology: validity of approach, quality of data, quality of presentation

• Hi-C data has been used to investigate the structure of bacterial chromosomes for several species. As such, the approaches taken by these authors seem valid. Nonetheless, based on my interpretation of the data in this and other Hi-C studies of bacterial genomes, it does appear that the dynamic range of signals shown on the interaction map (Figure 1a) are much lower for M. pneumoniae than found in other studies. In Figure 1a, the colors shown on the contact map, from blue to red, indicate the log2 contact frequency for each M. pneumoniae interaction bin. The colors correspond to a range from 212 to 212.9. If I am interpreting this correctly, that means a range of less than two fold for the signal differences. The numbers shown for Hi-C interaction maps of C. crescentus, B. subtilis, and E. coli vary from 50 to over 1000 fold. Can the authors explain why the M. pneumoniae interaction data appears to be so much less dynamic? As noted in the text, the interaction patterns in figure 1a are similar to those of B. subtilis and C. crescentus but the range is much smaller.

The maps for *C. crescentus* in our original manuscript were taken from Umbarger et al. 2011 and do not show normalized data, but rather Z-score data for the matrices. There the change of the data actually affects the scale of the differences by first applying a log scale and then calculating the Z-scores of the log values. As such, these two scales are not comparable anymore. In contrast, the work by Le et al. in Science showed actual normalized Hi-C interaction scores, which were normalized in a similar manner as ours (by using the ICE algorithm published by the Mirny Lab). Their resulting matrices were then further normalized so that the Hi-C scores for each row and column sum to 1. As seen in the plot below, when applying this additional normalization to our Hi-C replicates, by simply dividing the matrix by the mean of the sum of its rows, we obtained a very similar matrix.

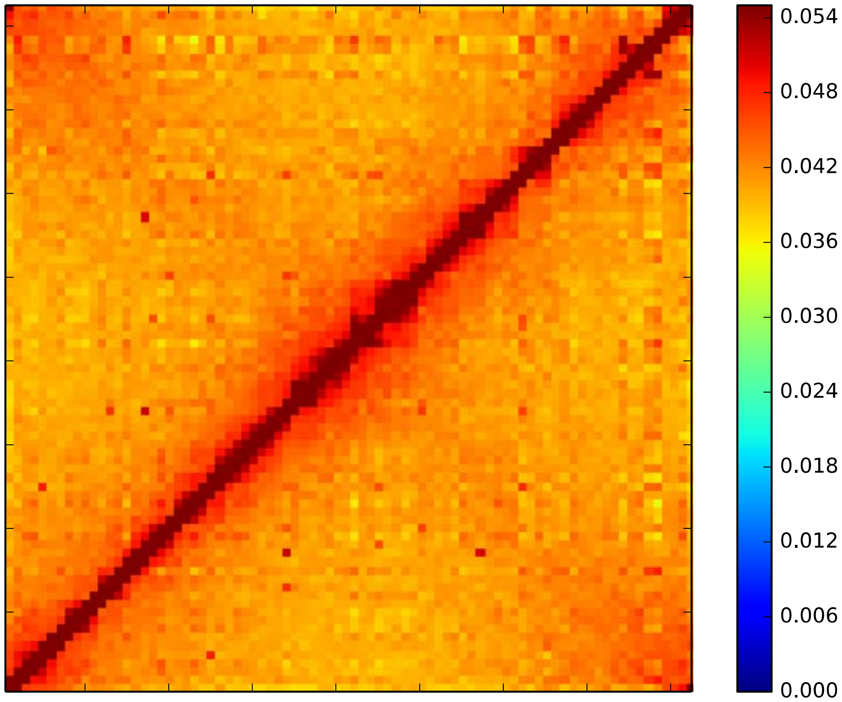

The correlation with the interaction map (Figure 1a) is 0.99 (p-value < 2.2e-16), and the Hi-C scores obtained are comparable to the Hi-C interaction maps of *C. crescentus* and *B. subtilis*. Therefore, the difference in the scale between previously published datasets and our dataset is due to the treatment of the data and does not affect the modeling of the data nor the conclusions we obtain from the analysis.

There should be data in supplementary information from the analyses of local interactions that partitioned the genome into 44 CIDs and then identified patterns of gene co-expression for genes inside a CID relative to genes in different CIDs.

We agree with the reviewer and have now provided a folder containing all the data and scripts used to reproduce the analysis (Coexpression_CIDs_borders):

1. Matrices_for_TADbit: contains the input raw and normalized matrices of the sum of the five HpaII replicates described in Figure 4a, of the HpaII with novobiocin described in Figure 5a, and of each of the five HpaII replicates, to calculate the CIDs borders.
2. Script_TADbit: contains the python script, iPython notebook script and html version, used in TADbit to detect the CIDs borders, and is saved in the Output_CIDs_detected/ directory.
3. Coexpression_Data: contains the two co-expression matrices (basal and Pearson) with their corresponding genes, as well as the co-expression for all genes pairs to compute the co-expression per CIDs.
4. Script_coexpression_CIDs: contains the R script used to compute the absolute mean co-expression of pairs of genes within and between CIDs.

• **The sequence data should be posted in a data repository.**

The accession number details for the reviewers to have full access to all of the Hi-C generated data used in our manuscript is E-MTAB-3721 at the https://www.ebi.ac.uk/arrayexpress/experiments/E-MTAB-3721/ with username: Reviewer_E-MTAB-3721 and password: RHG24ddR.
The RNA-seq data is already public with accession numbers: E-MTAB-3771, E-MTAB-3772, and E-MTAB-3773.

• **Lines 745-9. Co-expression tendency based on RNA-seq expression over 282 conditions. The meaning of this statement as well as the whole topic of co-expression is unclear. The experiments that report.**

Junier et al. measured the transcriptional activity of 869 *M. pneumoniae* genes across 141 conditions (282 samples). To analyze the level of basal coordination of transcription between all pairs (i,j) of genes, they developed a specific measure of transcriptional co-expression levels that quantifies the tendency of the expression of two genes to systematically vary in parallel. More specifically, it determines the difference between the number of pairs of samples for which the two genes co-vary and the number for which they vary in the opposite direction, normalized by the total number of possible pairs of samples. Compared to other related correlation measures, such as the Pearson correlation or the biweight midcorrelation, this technique is particularly well suited to highlight the basal coordination of genes, irrespective of the large shifts in expression levels that may occur in some of the conditions.

We performed analyses of co-expression tendency using the co-expression matrices from both the Pearson correlation and the basal correlation. Since both matrices led to the same findings, we only presented in the manuscript the results based on the Pearson correlation. However, as mentioned above, we have now provided the two co-expression matrices along with the script used for the analysis.

• **Furthermore, the in press Junier paper that was listed should have been provided if it was necessary to reference it.**

We agree with the reviewer and we provide the manuscript now published in Cell Systems.

D. Appropriate use of statistics and treatment of uncertainties. I am not qualified to judge this. I can say that the authors largely used some of the same statistical tests employed by other researchers that have analyzed Hi-C data from bacteria. However, it is by no means clear how many of the statistics they report were calculated or what the statistical tests are actually measuring.

In this study, we tested several hypotheses using p-values. If the p-value is less than (or equal to) α, then the null hypothesis is rejected in favor of the alternative hypothesis. On the other hand, if the p-value is greater than α, then the null hypothesis is not rejected. In most studies, authors refer to results being

statistically significant when p< 0.01 or p< 0.05 and being statistically highly significant if p< 0.001.

The first statistical test we used was to compute the correlation between Hi-C replicates and is based on Pearson's product-moment correlation coefficient. It is a measure of the linear correlation between two variables. The five HpaII biological replicates have a correlation of r> 0.91 with a highly significant p-value< 0.0001. Similarly, the correlation between the HindIII and HpaII datasets is r> 0.81 with a p-value< 0.0001.

The second test we used was a permutation test to assess whether a given factor, such as the number of HpaII sites, the GC content, the number of convergent and divergent genes, or co-expression levels, is related to the domain borders. We performed the permutation test by shifting all domain border positions across the entire genome, while conserving both the size and number of genomic domains. Then, for each permutation, we calculated the mean factor number at the domain borders. Finally, we computed the empirical p-value as the ratio between the number of values that are lower/higher than or equal to the observed value in the original domain border case. For example, we found a significant number of both convergent and divergent genes pairs with p-values= 0.026 and 0.037, respectively.

The third test we used was the two sample t-test to test whether the absolute mean co-expression of pairs of genes within and between domains are equal. A p-value of 0.0032 was obtained, indicating a true difference in means. Therefore, we were able to conclude that genes are significantly co-expressed within domains.

E. Conclusions: robustness, validity, reliability. For the most part the conclusions are consistent with the results. I did not find the argument that the dearth of chromosome associated proteins encoded by M. pneumoniae meant that the folding of the DNA was not mainly driven by proteins.

The point we wanted to make is that the total copy number of known structural proteins is proportionally smaller in *M pneumoniae* compared with other bacteria, and therefore its chromosome structure can be maintained using a reduced amount of such proteins (Supplementary Table 1). Of course, we cannot discard the possibility that other unknown, or mycoplasma-specific structural proteins are present. We have changed this point in the manuscript to reflect these thoughts.

F. Suggested improvements: experiments, data for possible revision
• Ideally the authors would insert a large segment of stuffer DNA in either the left of right half of the genome to determine whether the terminus of genome replication is at specific sequence or rather, just the spot on the chromosome that is approximately halfway from the origin. This could be done using a transposon with a large gene free stuffer, perhaps 50 or 100 kb, or direct recombination of DNA into M. pneumoniae has been reported (Krishnakumar, et al. 2014 PMID: 24914053).

We appreciate this reviewer for suggesting a complementary analysis. The paper mentioned by the reviewer (Krishnakumar et al., 2014) uses the cre-lox system to invert or delete genomic regions. However, to the best of our knowledge, this technique has not yet been described to be functional in *M. pneumoniae*. In

Krishnakumar et al., 2010, it was shown that this process has a very low efficiency in the MPN129-B7 strain that we are using (only one colony was obtained for this strain). Indeed, it does require a double cross-over recombination of DNA. Also, in our hands we have not been successful at getting homologous recombination to work using the endogenous machinery of the cell.
The other alternative is to use a mini-transposon, but different fragment sizes and combinations would be required, as the region of the terminus has not yet been defined in *M. pneumoniae.* Moreover, inserting a large region of 50-100 kb is challenging, and so far, we have only been able to achieved a maximum insertion of 10 kb.

In any case, we agree with the reviewer and have deleted the word ´Ter´ since it is misleading and we don't have functional evidence proving that this region is the real Ter. We now mention that a region approximately opposite to the Ori is found near the attachment organelle, and now refer to it as the ´midpoint´ of the chromosome. The exact localization of the Ter is not essential for our study and does not affect any of our conclusions.

• The authors note that M. pneumoniae has few genes whose products might be expected to take part in establishing chromosome structure. On line 405 they write: As M. pneumoniae however has a limited number of copies of the histone-like IHF protein, thereby making it difficult to maintain the CID boundaries, it is likely that additional factors contribute to the formation of such domain loops. Furthermore, since M. pneumoniae only has a handful of DNA-binding proteins and very few TFs (Table 1), it is intriguing that it is capable of establishing a well-defined chromosome structure as well as maintaining CID boundaries. The list of M. pneumoniae transcription factors, sigma factors, and structural proteins (Table 1) should also include how many copies of each protein are present on average. These authors have determined this in other work. If they are adamant about this theory they could even report how many copies of each protein exist per basepair of DNA and how many exist in the organism they were comparing M. pneumoniae with, C. crescentus.

We agree with the reviewer that comparing the copy number per base pair of *M. pneumoniae* with other bacteria is a more accurate assessment. As such, in Supplementary Table 1 we provide a list of DNA-binding proteins with their average copy numbers per base pair of DNA for *M. pneumoniae* and also for *E. coli* and *B. subtilis*.

Interestingly, some *M. pneumoniae* proteins like gyrases and topoisomerases have a higher copy number per base pair compared to those of *E. coli* and *B. subtilis*. However, the total non-transcription factor DNA-binding protein copy number normalized per base pair for *M. pneumoniae* is 0.001, while for *E. coli* and *B. subtilis* it is 0.01. Altogether this additional analysis not only highlights that *M. pneumoniae* lacks several structural DNA-binding proteins but also demonstrates that the total number of copies of its remaining proteins is limited when compared to *E. coli* and *B. subtilis*. Despite this, and as mentioned above, we cannot rule out

the possibility that other specific mycoplasma proteins play a structural role. There, we have toned down the related comment in the manuscript.

G References: appropriate credit to previous work?
Yes

Clarity and context: lucidity of abstract/summary, appropriateness of abstract, introduction and conclusions

This was a difficult paper to read and understand. In its current form, it should not be published. I do not think of Nature Communications as a specialty journal. Accordingly, a reader needs to be able to understand the data that are presented without needing to invest in reading still another paper in order to appreciate and evaluate the new paper. Furthermore, the reason that a paper on an otherwise esoteric organism that is a minor human pathogen is under consideration for Nature Communication publication is because M. pneumoniae is a model for a minimal bacterial cell. The paper needs to be written as more of a story than it is now. The only way I was able to get through it was to read very carefully the Hi-C of Caulobacter cresentus paper by Umbarger, M.A. et al. The three-dimensional architecture of a bacterial genome and its alteration by genetic perturbation. Mol. Cell 44, 252-64 (2011).

We apologize to the reviewer for the lack of clarity and we have tried to make it clearer in the new version.

The authors use, but do not define, the term co-expression. In discussion of the phrase with my colleagues, some thought that genes in a single transcriptional unit are co-expressed. Others thought that co-expression pairs of genes transcribed at similar levels. The term needs to be described clearly. Furthermore the data used to calculate co-expression levels needs to be included in supplementary materials.

We agree with the reviewer and, as previously mentioned, now make available the two co-expression matrices (basal and Pearson) with their corresponding genes, and the script and co-expression for all gene pairs used to compute the co-expression per CIDs.
In the manuscript of Junier et al. (now included in our response), the term co-expression refers to the co-expression between all pairs (i,j) of genes, for the 869 *M. pneumoniae* genes across 141 conditions (282 samples). This quantifies the tendency of the expression of two genes to systematically vary in parallel. This has now been clearly defined in the manuscript.

Figure 1. The last part of the Figure 1 legend states: "The color indicates the strand position, with pink being the - strand and green the + strand. (c) 3D density map representation of the first cluster of M. pneumoniae genome

models with Ori and Ter represented by red and purple circles, respectively. A color tube shows the centroid model, following the same color code as the bar in (a), and the lighter color represents the space occupied by all the models in the cluster, i.e. the variability across the cluster." There is a lighter shading of the figure below the origin position. Does that indicate the model predicts the absence of chromosomal DNA in that region of the cell. If so why, I thought the terminal organelle was nearer the terminus. Also, the sentence that begins with "A color tube shows the centroid model..." I presume this is jargon to explain that the line represents the highest probability model for chromosome location. In any case, the sentence is not clear in its meaning.

> Figure 1c is a 3D representation of 516 superimposed model structures from the first cluster of *M. pneumoniae* genome models. A central model, referred to as the centroid, is the model that is closest to the mean x,y,z coordinates compared with all the other models. This centroid is shown as a colored tube, starting with particle 1 in blue and ending with particle 82 in red - the same color code that is used in the bar in Figure 1a. The Ori and midpoint particles are highlighted with red and purple circles respectively.
>
> The lighter color is an “artefact" of the visualization software used for preparing the figure (Chimera software from UCSF). Chimera uses a “lighting” scheme to create a sense of 3D in the image. What appears to be a lighter patch is in fact the lighting imposed by the software in the parts of the image that are closer to the light source (which is by default the upper-left side of the screen).

Figure 4 is confusing. The legend mentions that the data in 4a is filtered and normalized. In fact at six sites the paper the data is reported as being filtered and normalized. The authors reference the Imakaev et al. paper "Iterative Correction of Hi-C Data Reveals Hallmarks of Chromosome Organization" however the process is presented as a black box. There should be an explanation of why this is done and how it changes the outcome of the analysis. The 4a diagram has domains represented as grey-filled arcs delimited by a colored line. There is no explanation why 15 of the arcs are light grey and 29 are dark grey. Because the authors felt it was necessary to show the Hi-C matrix 3kb res data (the blue section of the figure), there must be data that needs to be gleaned from the diagram, but I cannot discern what that might be. In 4c, the authors state "Detailed absolute mean co-expression distribution across the 44 domains. Point sizes are proportional to border strength. The color depicts, as before, the two cases of gene pairs within the same domain, shown in green, and gene pairs between different domains, shown in blue. " From this data, I am led to believe that at least on one side of each domain, there is a transcriptional unit with at least one gene in each domain. Otherwise, some of the domains would be represented by only green dots. Am I misinterpreting this? Finally, in part 4a Hi-C matrix interact. Freq. can be as high as 1.5 apparently. While in 4b, c, and d it is only possible to achieve a level of 1.0. These y-axis values have unclear units of measure. Interpretation is not easily possible.

We agree with the reviewer and have included detailed explanations of the normalization and filtering in the revised version of the manuscript.

To construct the interaction maps, read pairs of 50 bp were uniquely mapped to the MPN129 reference genome (NC_000912, NCBI) covering 816,394 bp. This was done using Bowtie2 and following the ICE iterative mapping strategy from the hiclib Python (Imakaev et al.). The iterative mapping procedure starts with a read length of 25 bp and increases by steps of 3 bp until a maximal read length of 40 bp is reached. Only read pairs for which both reads uniquely aligned to the genome were considered in subsequent steps. The MPN129 genome was divided into restriction fragments (449 HindIII fragments, 1,411 HpaII fragments) and each read of a read pair was sorted into its corresponding restriction fragment. Read pairs were classified as valid Hi-C products, non-ligation products, or self-ligation products and with only the valid Hi-C products were considered. We then constructed a genome wide matrix, M, of different resolutions (3, 5, 10, 15 and 20 kb) by dividing the genome into 3, 5, 10, 15 and 20 kb bins, and pooling interactions into their corresponding bins. Essentially, the number of interactions, or read counts was converted into Hi-C scores by applying the following equation and iteratively repeating it for the resulting contact map after each cycle: mij =mij * (total reads) / (total reads in bin i * total reads in bin j). The iterative procedure was repeated until the maximum relative error of the total number of Hi-C scores in a bin was less than 10^{-5}. Additionally, the MPN genome contains a considerable number of DNA repeats. Those DNA fragments having a high sequence similitude could hybridize and be found in the library of interacting fragments. Therefore, we performed a Hi-C control library with no formaldehyde fixation to remove any possible artifacts derived from the library preparation. When generating the Hi-C maps, we then added a filtering step using this control library to exclude those interactions higher than the second diagonal, which are not due to 3D contacts in the chromosome and were found in both samples. Therefore, these filtered interactions are excluded from all analyses.

In Figure 4a, each grey-filled arc represents a CID. The height of the domain is proportional to the relative amount of interactions in the domain given its size. The black horizontal line highlights the expected number of interactions given the domain size. If the Hi-C relative interaction frequency inside the CID is higher than 1, that is, higher than expected according to its size, then the domain is colored in dark grey.

In Figure 4c, we are plotting the absolute mean co-expression across the 44 domains. As previously explained, co-expression refers to the degree by which genes change in the same direction under different perturbations, between all pairs (i,j) of genes. Here we compared the absolute mean co-expression of pairs of genes within and between domains. There is a median number of 240 pairs of genes within domains and 13,608 pairs between domains. For the case of gene pairs within the same domain (shown in green), co-expression was computed for all gene pair found in the same domain. In the second case (shown in blue) it was computed for all gene pair found in two different domains.

Finally, as previously explained, in the y-axis of Figure 4a we are plotting the relative frequencies of the Hi-C interactions of each domain. In contrast, in the y-axes of Figures 4b, c, and d, we are plotting the absolute mean co-expression level across the domains. As such, these two different y-axis values are not comparable.

Figure 5 has many of the issues listed above for Figure 4. One specific question is what is CID density, what are the units, and how is it calculated.

The CID density is computed as the sum of the Hi-C interactions in a domain divided by the number of expected interactions. The number of expected interactions is computed as in Rao et al. 2014, where for each genomic distance an average number of interactions is calculated. The units of CID density are thus interactions normalized by the genomic distance.

Figure 6 shows four different models for bacterial chromosome organization. The authors state the M. pneumoniae model was generated by TADbit software. I think this was the same software used at least for the C. crescentus modeling. Ideally, the models should be drawn so as to be more comparable. It is unclear how much of what is shown is an artistic interpretation of chromosome architecture and how much is a visual output from the TADbit software.

TADbit software does not produce any representation of the models, in fact, it results in the x,y,z coordinates for all modeled particles. The “artistic” interpretation is produced by the Chimera Software developed by Prof. Ferrin at UCSF (UCSF Chimera--a visualization system for exploratory research and analysis. Pettersen EF, Goddard TD, Huang CC, Couch GS, Greenblatt DM, Meng EC, Ferrin TE. J Comput Chem. 2004 Oct;25(13):1605-12.). Actually, we also used this software in our previous work (Umbarger et al), and have adapted the legend to reflect this.

Reviewer #2 (Remarks to the Author):

Bacterial chromosomes are now known to be highly organized and potentially serve biological roles. Chromosome organization were widely studied in bacterial model organisms such as E. coli, B. subtilis and C. crescentus. However, the organisation in other lesser-known bacterial species have not been explored. In this manuscript, Trussart et al employed latest technologies such as chromosome conformation capture (Hi-C) and high-resolution FISH to explore the chromosome organization of a genome-reduced Mycoplasma pneumoniae. Luis Serrano and Marc Martin-Renom are experts at systems biology of M. pneumonia and at computational analysis of Hi-C data, respectively. I was eager to read this manuscript but in the end I found this manuscript immature for Nature Communications. There are not much experimental data but the authors made very bold statements.

We would like to address the reviewer´s concerns in the text. We respectfully disagree with the reviewer in saying that there is a limited amount of data and analysis. It is important to note that we have used three different experimental approaches to determine the 3D structure of the *M. pneumoniae* genome. We not only performed Hi-C experiments with two different enzymes, but also performed two additional experimental analyses with electron microscopy and super-resolution imaging, in order to compare with Hi-C data and further validate the chromosome conformation.

Further analyses were done using a 3D restraint-based modeling approach to determine the folding of the *M. pneumoniae* chromosome. Additionally, we performed domain detection analysis to assess whether this reduced genome is divided into domains. Finally, we combined the previously mentioned domain analysis with a co-expression analysis of RNA-Seq expression data over 282 conditions to provide evidence that genes inside CIDs tend to be co-regulated. This co-regulation suggests that chromosome organization influences transcriptional regulation. Moreover, to further relate CID domains to transcription, we studied the effect of supercoiling inhibition on chromosome structure by performing Hi-C on cells treated with novobiocin, an inhibitor of the gyrase protein.

We therefore believe that this is probably the most comprehensive study of the structure of a bacterial chromosome.

Below are my specific points:

1. Abstract page 2 line 32-33: the authors wrote "DNA-binding proteins are central regulators of chromosome organization, however in genome-reduced bacteria their diversity is largely diminished" and they keep using this to argue that "nucleoid-associated proteins are not essential for chromosome organization Abstract page 2 line 42-44". Looking at the Table 1, clearly this is not the case. M. pneumoniae has SMC, Ihf, CbpA together with DNA

topoisomerases. This is about the same number of NAPs that B. subtilis and C. crescentus has. Indeed, this is nowhere near the number of NAPs in E. coli but it could well be that new and important classes of NAPs have not been discovered yet. There are more in E. coli just because it is a very well-studied bacterium.

> We have already addressed this issue in our response to Reviewer comment 1F. We now provide a list of DNA-binding proteins with their average copy numbers normalized per base pair of DNA for *M. pneumoniae*, *E. coli* and *B. subtilis* in Supplementary Table 1.
> Interestingly, some *M. pneumoniae* proteins like gyrases and topoisomerases have a higher copy number per base pair compared to those of *E. coli* and *B. subtilis*. However, the total DNA-binding protein copy number per base pair for *M. pneumoniae* is 0.001, while for *E. coli* and *B. subtilis* it is 0.01. Altogether this additional analysis not only highlights that *M. pneumoniae* lacks several structural DNA-binding proteins but also demonstrates that the total number of copies of its remaining proteins is limited compared to *E. coli* and *B. subtilis*. We have added a paragraph indicating that we are unable to rule out the possibility that other unknown mycoplasma-specific proteins compensate for the low overall copy number of known structural DNA binding proteins.

2. M. pneumoniae indeed has a reduced genome but they have as many NAPs (see point 1 above). That can only argue that NAPs, SMC and topoisomerases are very important (potentially for chromosome organization), so important that even genome-reduced organisms must keep them. Therefore, I don't agree with the sentence "nucleoid-associated proteins are not essential for chromosome organization Abstract page 2 line 42-44". Furthermore, in C. crescentus and B. subtillis, when smc is deleted, the less prominent secondary arm is gone suggesting that SMC is needed for a global chromosome organization. Deletion of hup again sees the reduction in short-range interactions. Perhaps, the authors should delete or deplete smc/ihf/cbpA in M. pneumoniae before concluding that they are not important for the chromosome organization in this species.

> We agree with the reviewer and have toned down our conclusions and addressed them in the revised manuscript.
> SMC, Ihf and CbpA are classified as essential and strong fitness proteins in the essentiality study that was previously published (Table 1, Lluch-Senar, 2015). This fitness category refers to genes that have an impact on growth and whose essentiality depends on conditions and transposon insertions. Unfortunately, we have not been able to isolate a specific mutant from the transposon mutant libraries. Furthermore, targeted depletion of such proteins requires double-recombination, a process which has an extremely low efficiency in *M. pneumoniae* (Krishnakumar et al., 2010). In any case, as mentioned above, we have toned down this discussion in the revised manuscript.

3. The authors seem to define the chromosome organization as "having domains and domain boundaries". This is just one feature of the chromosome structure and it is still too early to be certain that domains are

of biological significance.

As mentioned by the reviewer, we were able to show that a reduced genome is partitioned into structural domains. Although it has not yet been extensively proven that domains modulate biological function, we do provide here various evidence that genes inside CIDs tend to be co-regulated, thereby suggesting that the presence of such domain influences transcriptional regulation.

4. Result Page 7 line 134 "Although the Hi-C interaction maps obtained at exponential and stationary phase display similar features, the analysis of the chromosome structure at exponential phase could be hampered by heterogeneity, as it is not possible to synchronize M. pneumoniae. Therefore, we concentrated on the stationary phase samples". I do not agree that the Hi-C maps for exponential and stationary phase are similar at all. Looking at Supp figure 5, I don't see the less prominent secondary diagonal. It looks more like a failed Hi-C since there is nothing coming out from the background noise except for the primary diagonal which is very strong in any case anyway. The correlation between exponential phase and stationary phase samples is 0.6 (not that strong) and potentially most of the 0.6 is from the strong dominant diagonal. Could the authors revise the way they calculate the Pearson's correlation value please? Specifically, I would like them to block away the main diagonal and just compare the rest of the Hi-C maps between the two conditions. Also, please report how many reads are discarded in each step of the Hi-C analysis so readers can judge if this is indeed a failed ligation step, leading to a failed Hi-C. Right now, the authors only report the start and the final number of reads.

We agree with the reviewer and have added detailed information of each filtering step in the revised manuscript.

We performed two additional Hi-C experiments at exponential phase (three HpaII replicates in total) and computed an interaction matrix as the sum of the three replicates. This was done at a 10 kb resolution and normalized by the respective number of reads for subsequent analysis. This is now plotted in the new Supplementary Fig. 5.

We obtained a Pearson correlation of 0.72 between exponential and stationary samples, and a correlation of 0.46 when computing only off-diagonal interactions. We do not observe the less prominent secondary diagonal in any of the three replicates, or in the computed matrix sum.

As the total number of reads in stationary phase is significantly higher, we performed an additional subsampling analysis of the five replicates obtained for this phase to ensure that the difference in the two matrix profiles is not related to the number of reads. Therefore, we subsampled the raw Hi-C data of the stationary phase by randomly selected a total number of reads that is equal to the total number of reads obtained in the raw Hi-C data of exponential phase.
Interestingly, we still observe a less prominent secondary diagonal in the subsampled Hi-C replicates of the stationary phase, and indeed the mean

correlation between the subsampled replicates in stationary phase (0.82) is significantly higher than those in exponential phase (0.65), plotted below.

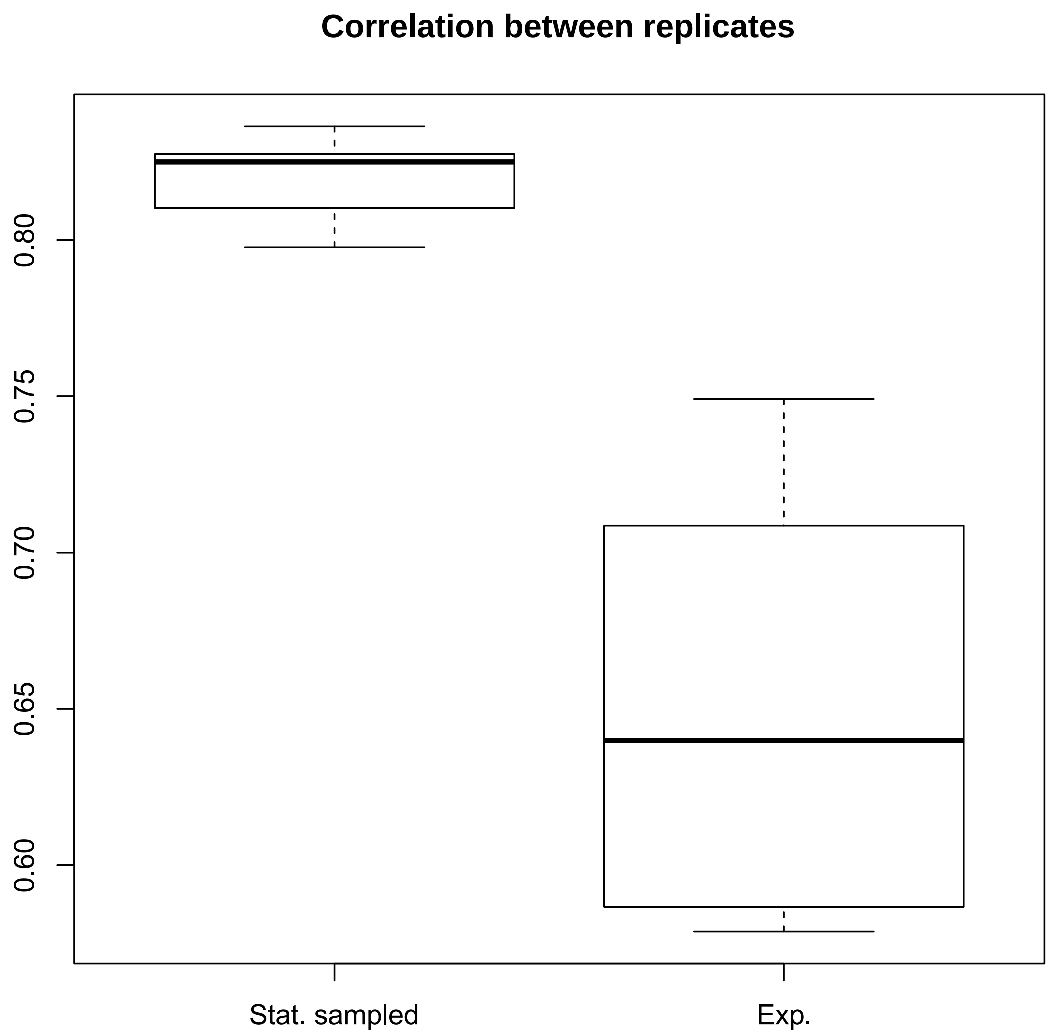

In any case, we have now performed FISH experiments at stationary phase and consider both FISH and Hi-C at stationary phase in the revised manuscript. Additionally, we keep the exponential phase FISH distances and comment on them in the text.

5. I have just looked at the Le et al 2013 Caulobacter Hi-C paper again and it seems that even in a mixed population of Caulobacter crescentus (un-synchronized), all the features of the maps (main and secondary diagonals) are still seen so I don't think that the not-so-good exponential phase map of M. pneumoniae is due to too much heterogeneity.

There is a fundamental difference between *M. pneumoniae* and *C. crescentus* with respect to division time. *M. pneumoniae* duplicates, at its best, every 8 hours while *C. crescentus* does so every 90-150 min. Moreover, there are also suggestions that DNA replication in Mycoplasma could be quite slow (Seto and Miyata, 1997). Together, both of these features could result in significant heterogeneity in the population regarding chromosome structure. In any case, we have now repeated the Hi-C experiment at exponential phase two additional times and observe the same heterogeneity across the new replicates as previously explained.

6. I am especially worried about using stationary phase to do Hi-C and to generalize about chromosome organisation. It is known that in E. coli, stationary cells have especially compacted chromosome/nucleoid, very different from exponential phase. Furthermore, the authors use exponential phase cells (instead of stationary phase cells) for FISH microscopy so it is just not possible to merge the two datasets to say a concrete and consistent thing about the chromosome organization in M. pneunmoniae.

We agree with the reviewer and have performed new FISH experiments at stationary phase to be able to compare with the Hi-C dataset obtained in this same phase. The distances obtained from cells in stationary and exponential phase are similar (Supplementary Figure 5). Thus, although we were are unable to reconstruct the chromosome in exponential phase probably because of structural heterogeneity, it seems they are not so different.

7. Measuring the nucleoid volume of DAPI stained cells is a weak way to validate the model. The model does not give us much more information than the FISH data. Just by looking at the FISH data, one can already learn that ORI and TER are at the 2 opposite poles of the cells and LEFT and RIGHT are in the middle. Interestingly, the 2 arms of the chromosome in M. pneumoniae are not twisted like in the case of C. crescentus. I believe the same group did the modelling for C. crescentus as well. This is an interesting point that could be explained by combining modelling with more FISH data of more DNA loci. Perhaps this will strengthen the manuscript?

We agree with the reviewer and have performed an additional experiment using 3D-sim technology and DAPI staining to more precisely estimate the 3D nucleoid volume. This additional estimation has been added in the revised manuscript.

To further validate our model we have also performed new FISH experiments by marking three additional regions (N1, N2 and N3 probes). These new distances also agree with the 3D model, thus confirming a lack of twisting. Secondly, we have repeated some of the measurements on another localization microscope system (Nikon N-STORM) in 3D and obtained similar results as illustrated for the midpoint-AO estimation in Supplementary Fig 9.

We compared the cell volumes of *M. pneumoniae* and *C. crescentus*, 0.075 μm^3 and 2.36 μm^3 respectively, considering a cylinder of 0.5 µm in length and 3 µm in height for *C. crescentus* cells. Moreover, we observed a difference in the ratio of genome length to cell volume (bp/μm^3) when comparing the two genomes. This

ratio is 1.5 higher for *C. crescentus,* indicating that its genome might be more compact than the genome *of M. pneumoniae*. This might also reflected by the twist observed in its chromosome structure.

8. Figure 4. I can hardly see the domains and domain boundaries at all. The colour choice makes it really hard to see. Perhaps the authors should present it better?

We agree with the reviewer and this has been addressed in the revised manuscript.

9. I don't agree that genes within each domain are co-regulated. Domain size are 15-30kb in this study, much longer than an average operon in bacteria. That makes the co-expression analysis within versus between domain faulty to start with. In this case, the co-regulation of genes is dominated by operon structure. It has little to do with chromosomal domains.

Operons in mycoplasma are small and the majority of the genes are monocistronic or bicistronic. There is a total of 671 operons and 852 suboperons in *M. pneumoniae* with a median size of 605 bp and a maximum size of 9,197 bp. In comparison, the domains identified in our study have a median size of 15,000bp. We find on average 15 operons and 18 suboperons per domain, and our analysis shows that these different operons tend to be more co-regulated than operons located in different domains.
As an illustration of the distribution of operons across domains, we plotted in Supplementary Fig.7 the suboperons found in CIDs 8 and 9, which have a total number of 20 and 14 suboperons, respectively.

Reviewer #3 (Remarks to the Author):

The MS studies 3D genome structure in a small bacterium. This is a highly interesting scientific problem: Ample evidence has shown that not only mammalian cell nuclei but also other vertebrates and non-vertebrates maintain a highly sophisticated 3D nanostructure of their genome which is supposed to play an important role in transcription, splicing, replication and repair. From the evolutionary point of view one might speculate that such structures might have started to be formed already at the beginning of life, due to the selection pressure correlated with functional spatial organization.

The authors address this problem by combining Hi-C and super-resolution microscopy, to determine the structure of the M. pneumoniae chromosome at a 10 kb resolution. From modelling Hi-C data, they found evidence for a defined structure, with a global symmetry between the two arms connecting the opposite poles bearing the chromosomal Ori and Ter. A more refined Hi-C analysis of local structures at a 3 kb resolution indicated that the chromosome was further organized into small subdomains, with a size from 15 to 33 kb. In eukaryotes, this would correspond to clusters with a number of nucleosomes in the range around 100 - 200. Since recent findings from localization based super-resolution methods suggested that small nucleosome clusters might exist also in eukaryotes, such a finding might have a great potential interest for a better understanding of the evolution of spatial genome structure. In addition, the authors found that genes within the same domain tended to be co-regulated,suggesting that even in minimal bacteria local chromosome organization influences transcriptional regulation. This influence has been well established in mammalian cells; its existence even in minimal bacteria supports the notion of a long evolutionary history. Altogether, the findings indicate that a defined chromosomal structure is a universal feature existing throughout evolution; even nucleoid-associated proteins appear to be not essential for this.

Specific remarks:

1) Hi-C modelling: Although for the general approach a number of quotations are given, it is not clear how these principles are applied in detail to the present data set. It is recommended to provide more detailed evidence for this in the supporting material; since in principle the Hi-C data provide interaction probabilities only, not directly spatial coordinates, the modelling and its implications are of great importance.

> We agree with the reviewer and this has been addressed in the revised manuscript.

2) The ms contains a fair amount of quite detailed conclusions read from the Hi-Ci map. While the description and basic evaluation of the Hi-C data appears to be plausible, these detailed conclusions are difficult to follow by a reader who is not a professional expert in the interpretation of such Hi-C

graphs. It is suggested to give a broader justification for each of these detailed conclusions. This can be done in the supplementary material.

We agree with the reviewer and this has been addressed in the revised manuscript.

3) Superresolution imaging: An obvious way to reduce the ambiguity inherent in the derivation of spatial structures from Hi-C data is superresolution imaging. These novel techniques (the development of some of these approaches was honored by the 2014 Nobel prize in Chemistry) allow to extend the range of light microscopic imaging to the single molecule resolution level. Publications e.g. by the Xiaowei Zhuang lab (see quotation 9) show how effectively in particular methods of localization microscopy can be used for the study of bacteria. Towards this goal, the authors so far made little efforts, compared with the broad claim given in the abstract: They did some experiments using a commercial localization microscope (GSDIM). The quotations (58,59) they give for the method cover some aspects of localization microscopy; however, they do not describe the method actually used: while the Rust et al. paper describes the use of pairs of molecules, the Betzig paper is based on photoactivation of specific GFPs; they also do not describe the 'blinking' based localization microscopy using FISH which was successfully introduced already a number of years ago. Concerning the problem of two-color localization microscopy, three-color based localization microscopy (using human cancer cells and photostable dyes) has already been shown to work successfully in the beginning of the 2000s by a German-Israeli collaboration, achieving an intranuclear optical resolution of few tens of nm. Concerning the localization microscopy (GSDIM) based FISH results, the authors appear to evaluate the distances only; by doing so, they did not exploit the possibility to obtain not only the distances in a range much below the conventional optical resolution of ca. 200 nm but also the size of the labeled Ori/Ter regions at the nanoscale. It is recommended to provide additionally a more thorough evaluation of their GSDIM data according to the state of the art, and to compare the results obtained with the spatial predictions of the Hi-C data. At the present state, the claim made in the abstract of "combining Hi-C and super-resolution microscopy" looks somewhat too strong. To justify such a claim, in a revised ms it should be very interesting to provide localization microscopy data of a few other gene regions; e.g., it should be possible with this method to experimentally verify the finding of the Hi-Ci data of the existence of 15 - 33 kb small clusters; what is the spatial extension of such clusters? Various approaches how to achieve this by localization microscopy have been published and might be useful.

We agree with the reviewer and have added more references describing the method used in the revised manuscript.

We have performed new FISH microscopy experiments at stationary phase to be able to compare with the Hi-C dataset obtained in this same phase (Figure 2). Also to further validate our model we provide localization microscopy data for three additional regions marked by FISH (N1,N2 and N3 probes).

Furthermore, we performed two additional experiments using two different localization microscopy approaches. First, 3D-sim technology and DAPI staining were used to more precisely estimate the 3D nucleoid volume.
Second, we repeated some of the measurements with another localization microscope system (Nikon N-STORM) in 3D and obtained similar results as illustrated for the midpoint-AO estimation (Supplementary Fig. 9).

4) Discussion: For the general reader, the ms should be become much more interesting if imbedded into the general discussion of functional genome architecture and its evolution. For example, the ms makes believe that the partitioning of the mammalian genome into topologically distinct units was discovered only recently using sequencing data. While these results are highly important and relevant, the general discovery of such distinct domains has been established by light microscopy already in the 1990s. It should be helpful to add a few remarks/quotations on this. Especially interesting should be to compare the results also with functional genome nanostructure in mammalian cells as the presently most studied system.

We agree with the reviewer and this has been addressed in the revised manuscript.

REVIEWERS' COMMENTS:

Reviewer #1 (Remarks to the Author):

The authors have done a terrific job addressing the comments from each reviewer. The manuscript is truly much better that it was when first submitted. I am OK with the paper being published as is; however I think the authors should address the following issue. The Venter Institute team reduced the genome size of Mycoplasma mycoides from ~1 Million bp to a genome half as large. That minimized cell with half of its original genes removed and presumably its 3D structure radically altered was essentially the same as wild type <. mycoides. The cell division cycle slowed from 1 hour to 2 hours. Additionally, the Venter team radically reorganized the gene order in one eighth of the chromosome and observed no alterations in phenotype. These findings seem inconsistent with the Barcelona team's contention that there is important 3-D structure in the genome of a very similar mycoplasma. I think this contradiction deserves comment in the discussion section of this paper.

Reviewer #2 (Remarks to the Author):

In general, I am happy with the rebuttal letter and the revised manuscript. However, there are two remaining points that I would like to see them addressed:

1) In my initial review, I wrote "Figure 4. I can hardly see the domains and domain boundaries at all. The color choice makes it really hard to see. Perhaps the authors should present it better?". And in the rebuttal letter from the authors: "We agree with the reviewer and this has been addressed in the revised manuscript.". However, figure 4 is still the same. I can hardly see any domain at all. May I suggest the authors select a segment of the chromosome and zoom in to show clearly domains and domain boundaries?

2) The title of the manuscript "Defined chromosome structure in a minimal cell" is too strong. May I suggest the authors to be more factual, for example "Defined chromosome structure in a genome-reduced Mycoplasma pneumoniae"?

Reviewer #3 (Remarks to the Author):

Reviewer #3 only passed along confidential comments to the editor but had similar concerns regarding data presentation as their fellow reviewers.

Comments from the reviewers are shown in **bold**

Responses to the comments are shown in plain text

Reviewers' comments:

<u>Reviewer #1 (Remarks to the Author):</u>

The authors have done a terrific job addressing the comments from each reviewer. The manuscript is truly much better that it was when first submitted. I am OK with the paper being published as is; however I think the authors should address the following issue. The Venter Institute team reduced the genome size of Mycoplasma mycoides from ~1 Million bp to a genome half as large. That minimized cell with half of its original genes removed and presumably its 3D structure radically altered was essentially the same as wild type <. mycoides. The cell division cycle slowed from 1 hour to 2 hours. Additionally, the Venter team radically reorganized the gene order in one eighth of the chromosome and observed no alterations in phenotype. These findings seem inconsistent with the Barcelona team's contention that there is important 3-D structure in the genome of a very similar mycoplasma. I think this contradiction deserves comment in the discussion section of this paper.

> We appreciate that this reviewer acknowledges the relevance of our revised work. We agree with the reviewer and have added this discussion in the revised manuscript.

<u>Reviewer #2 (Remarks to the Author):</u>

In general, I am happy with the rebuttal letter and the revised manuscript. However, there are two remaining points that I would like to see them addressed:

1) In my initial review, I wrote "Figure 4. I can hardly see the domains and domain boundaries at all. The color choice makes it really hard to see. Perhaps the authors should present it better?". And in the rebuttal letter from the authors: "We agree with the reviewer and this has been addressed in the revised manuscript.". However, figure 4 is still the same. I can hardly see any domain at all. May I suggest the authors select a segment of the chromosome and zoom in to show clearly domains and domain boundaries?

> We apologize to the reviewer for the lack of clarity and we have tried to make it clearer in the new version, adding a zoom section with several domains and domains boundaries.

2) The title of the manuscript "Defined chromosome structure in a minimal cell" is too strong. May I suggest the authors to be more factual, for example "Defined chromosome structure in a genome-reduced Mycoplasma pneumoniae"?

We agree with the reviewer and have suggested three revised titles that might more accurately reflect the data presented in the manuscript.

- “Defined chromosome structure in a genome-reduced bacterium, *Mycoplasma pneumoniae*”
- “Defined chromosome structure in *Mycoplasma pneumoniae*”
- “*Mycoplasma pneumoniae*, a genome-reduced bacterium has a defined chromosome structure”.

Our preference is “Defined chromosome structure in a genome-reduced bacterium, *Mycoplasma pneumoniae*”, but we are happy if you prefer any of the other two.
